# A Compendium of Potential Biomarkers of Pancreatic Cancer

**DOI:** 10.1371/journal.pmed.1000046

**Published:** 2009-04-07

**Authors:** H. C. Harsha, Kumaran Kandasamy, Prathibha Ranganathan, Sandhya Rani, Subhashri Ramabadran, Sashikanth Gollapudi, Lavanya Balakrishnan, Sutopa B. Dwivedi, Deepthi Telikicherla, Lakshmi Dhevi N. Selvan, Renu Goel, Suresh Mathivanan, Arivusudar Marimuthu, Manoj Kashyap, Robert F. Vizza, Robert J. Mayer, James A. DeCaprio, Sudhir Srivastava, Samir M. Hanash, Ralph H. Hruban, Akhilesh Pandey

**Affiliations:** 1 Institute of Bioinformatics, International Technology Park, Bangalore, Karnataka, India; 2 Manipal University, Manipal, Karnataka, India; 3 McKusick-Nathans Institute of Genetic Medicine and Department of Biological Chemistry, Johns Hopkins University, Baltimore, Maryland, United States of America; 4 The Lustgarten Foundation for Pancreatic Cancer Research, Bethpage, New York, United States of America; 5 Dana-Farber Cancer Institute, Boston, Massachusetts, United States of America; 6 Cancer Biomarkers Research Group, Division of Cancer Prevention, National Cancer Institute, National Institutes of Health, Bethesda, Maryland, United States of America; 7 Fred Hutchinson Cancer Research Center, Seattle, Washington, United States of America; 8 Departments of Pathology and Oncology, The Sol Goldman Pancreatic Cancer Research Center, Johns Hopkins Medical Institution, Baltimore, Maryland, United States of America

## Abstract

Akhilesh Pandey and colleagues describe a compendium of potential biomarkers that can be systematically validated by the pancreatic cancer community.

## The Problem

### Do We Already Have Too Many Cancer Biomarker Candidates?

Although there is intense activity towards identifying novel biomarkers for cancers, especially those for early detection, it is not clear whether we already have too many biomarkers described in the literature. Given that there is no central repository of data pertaining to any cancer, it is difficult to estimate if we have too many proteins described as “potential biomarkers” for any cancer. Technological advances in genomics, transcriptomics, and proteomics have facilitated high-throughput studies in which the data are analyzed in isolation and a comparison with the published literature is not generally feasible for the entire dataset. A central repository will not only integrate all the information scattered across the literature, but will also serve as a reference for prioritizing and systematic testing of candidate biomarkers. Pancreatic cancer is a deadly disease for which the available biomarkers, such as CA19-9, lack the desired sensitivity and specificity for early detection [1]. Therefore, we took pancreatic cancer as a model and carried out a comprehensive literature survey to systematically catalog the overexpressed genes/proteins. Our objective was to develop a compendium of potential biomarkers that could be systematically validated by the pancreatic cancer community and serve as a prototype for similar efforts in other cancers.

## Strategy

**Figure 1:**
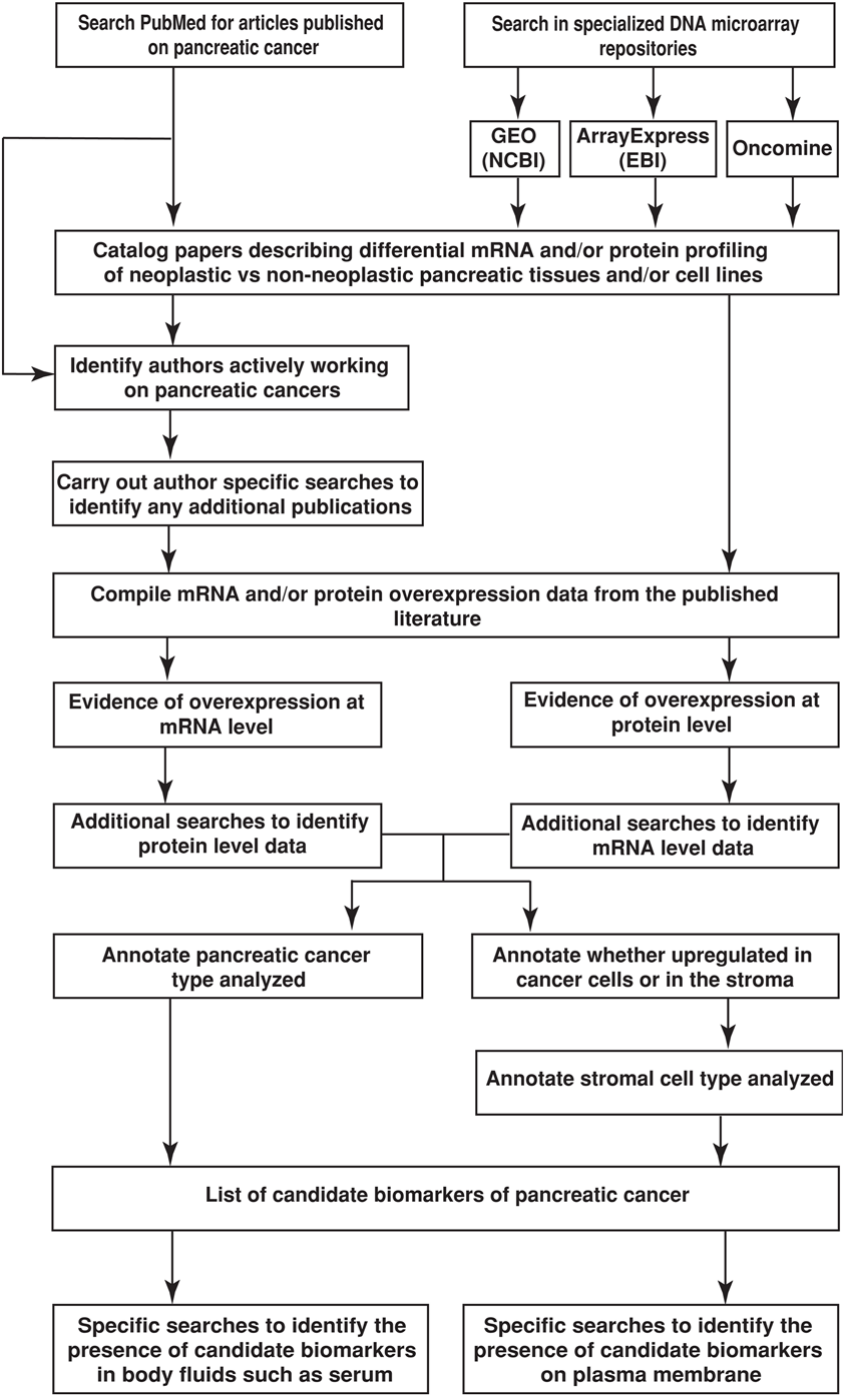
The curation protocol for generating the catalog of potential biomarkers. The curation protocol was entirely based on the published articles and data submitted to public repositories. Along with the list of molecules that are overexpressed in pancreatic cancers, literature evidence for their presence in plasma membrane and detectability in body fluids like serum was also searched for. The list includes overexpression studies from both endocrine and exocrine neoplasms of the pancreas.

With a large number of studies in the published literature (a keyword search for “pancreatic cancer” fetches ∼50,000 published articles), our approach (Figure 1) was to first identify the relevant publications and datasets (e.g., microarray data submitted to repositories such as GEO, ArrayExpress, and Oncomine [2]–[4]) that might contain information on overexpression of mRNAs or proteins in pancreatic cancer. The type of pancreatic cancer and the cell type where overexpression was observed were also annotated. After generating this list of molecules, specific searches were carried out to identify the presence of these molecules in body fluids or on the plasma membrane. Finally, queries were carried out to determine the status of these molecules in chronic pancreatitis, which is an important consideration in the differential diagnosis of pancreatic cancer.

### Inclusion Criteria for Candidate Biomarkers of Pancreatic Cancer

**Figure 2:**
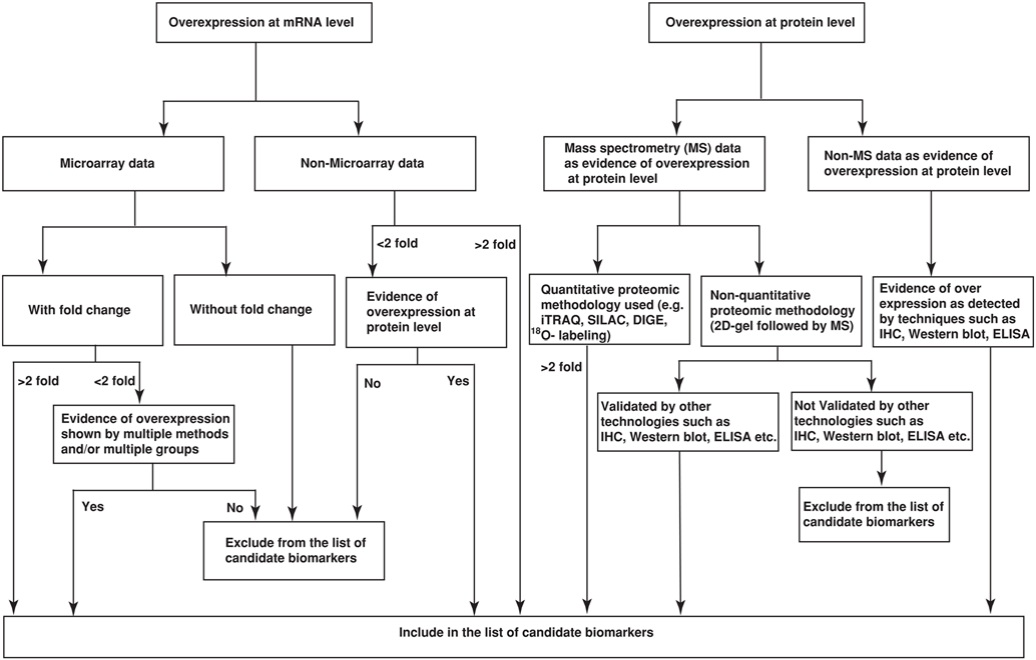
Criteria for inclusion as a potential biomarker. The primary criterion in compiling candidate biomarkers of pancreatic cancer was to identify molecules with an experimental evidence of overexpression either at mRNA or protein levels from the published literature. All molecules included in the list of potential biomarkers were required to have a minimum of 2-fold overexpression in cancer as compared to normal. Proteins reported to be overexpressed based on non-quantitative proteomic experiments were included only when there was additional evidence by alternative methods. Candidates from studies using antibody-based strategies like immunohistochemistry (IHC), ELISA, and Western blots were included without regard to fold changes, provided they were reported to be overexpressed in cancer as compared to normal.

Figure 2 shows the criteria for inclusion of molecules in this compendium. For an entry to be considered with evidence of overexpression at the mRNA level, there had to be a minimum of 2-fold overexpression in cancer as compared to normal samples. This is an arbitrary cut-off and was used mainly because it is the most common cut-off used by authors of such studies. DNA microarray data in which the fold changes were not specified were not included in the list. However, molecules that had a fold change of less than two were included if they were reported to be overexpressed by multiple methods. For the protein-level evidence, a protein was included if the quantitative proteomics was used (e.g., ICAT, iTRAQ, or SILAC methods) with a greater than 2-fold change. Molecules identified from non-quantitative proteomics methodologies (including 2-D gels) were included only if they were followed up with other techniques such as Western blotting, immunohistochemical labeling, or ELISA. Molecules without any such evidence were omitted from the list.

## Pancreatic Cancer Biomarker Compendium

### Summary of Molecules Overexpressed in Pancreatic Cancer

A total of 2,516 genes are included in this compendium, which includes molecules with evidence of overexpression at mRNA level, protein level, or both (see Table 1 for summary statistics). A total of 1,868 genes were reported as overexpressed only in the mRNA analyses, while 441 were reported as overexpressed both at mRNA and protein levels, and 207 molecules were reported as overexpressed only at the protein level. More than 200 genes are reported to be overexpressed in pancreatic cancers in four or more studies, making them excellent candidates for focused validation. Table A in Table S1 is a list of select molecules that are overexpressed in pancreatic ductal adenocarcinoma (PDAC, the most common type of pancreatic cancer), and that have been demonstrated by multiple methods at mRNA and protein levels. All molecules listed in this table are membrane-associated or secreted molecules and have immunohistochemical evidence, which makes them more reliable as potential biomarkers.

**Table 1:** Summary statistics of the pancreatic cancer biomarker compendium.

Feature	Statistics
Total number of published articles read	5,200
Total number of papers referenced in the final catalog	2,325
Total number of genes in the catalog	2,516
Number of genes with mRNA-level expression data	2,309
Number of genes with protein-level expression data	648
Number of genes with both mRNA- and protein-level expression data	441
Number of genes with only mRNA-level expression data	1,868
Number of genes with only protein-level expression data	207
Number of genes that have been tested on clinical samples by immunohistochemistry	386
Number of molecules that are both induced in pancreatic cancer and have been shown to be present in body fluids	930
Number of molecules that are both overexpressed in pancreatic cancer and have been shown to be present on the plasma membrane	567
Number of molecules upregulated in precursor lesions	1,094
Number of molecules overexpressed in cancer cells as well as stroma	266
Number of molecules overexpressed in stroma alone	5

### Secreted and Membrane Proteins Overexpressed in PDAC

For clinical screening of a potential biomarker, it is difficult to obtain tissue as a specimen since it requires an invasive procedure and a biopsy. Thus, it is desirable to have markers that could be detected in serum or other accessible body fluids such as urine. We cataloged studies in which the overexpressed molecules were detectable in body fluids by specific searches of the literature and from another community resource that we have recently developed called Human Proteinpedia [5]. The compendium provides a total of 162 secreted molecules that are overexpressed in pancreatic cancers at both RNA and protein levels. A select list of secreted proteins with multiple lines of evidence describing overexpression in pancreatic cancers at mRNA and protein levels, along with their detectability in body fluids, is provided in Table B in Table S1. Membrane proteins represent the majority of drug targets in use and are also attractive because of their potential use in cancer imaging and targeted therapeutic strategies. Also, plasma membrane-bound proteins are often shed into the bloodstream, which makes them useful candidates as biomarkers for early detection. Thus, focusing on membrane proteins overexpressed in pancreatic cancers could considerably enhance the chances of detecting and/or treating pancreatic cancer lesions at an early stage. The compendium lists 166 membrane molecules that are overexpressed in pancreatic cancers at both RNA and protein levels. A partial list of cell surface proteins with multiple reports of overexpression in pancreatic cancers at both mRNA and protein levels is provided in Table C in Table S1.

### Precursor Lesions

Pancreatic intraepithelial neoplasia (PanIN), intraductal papillary mucinous neoplasms (IPMNs), and mucinous cystic neoplasms are well-defined precursor lesions of invasive pancreatic cancer [6]. The genes overexpressed in these potentially curable precursor lesions would be ideal for early detection of pancreatic neoplasia. Global gene expression profiling studies using microdissected precursor lesions have revealed that there are several differences, but also commonalities, in gene expression between these precursor lesions and invasive PDACs [7]. Nearly 1,100 molecules have been reported to be overexpressed in PanINs and IPMNs. A large majority of these molecules show elevated expression in PDACs as well as precursor lesions. For example, molecules such as S100P, MMP7, MUC4, FSCN1, and MUC5AC are overexpressed in PanINs and IPMNs, as well as PDACs. All of them have been investigated by several methods including immunohistochemical labeling. Table D in Table S1 shows a partial list of molecules overexpressed in precursor lesions and their status in PDACs, if reported. As most of the protein molecules listed in Table D in Table S1 are detectable in body fluids, targeted studies to confirm the specificity of their expression pattern in precursor lesions and PDACs could provide early diagnostic markers with higher specificity and sensitivity. Particular caution should be taken while considering molecules observed in PanINs as early diagnostic markers of pancreatic cancer. While PanINs have been observed to progress into infiltrating carcinoma in some instances, it is not known how often or how rapidly they progress. Further, lower-grade PanINs (PanIN-1 and PanIN-2) are common in the older adult population, with a limited number of cases known to go on to develop cancer. Thus, biomarkers specific to PanIN-3 would prove useful as early diagnostic markers of pancreatic cancers. Continued efforts to study early stages of pancreatic cancer are therefore vital in identifying markers for early diagnosis.

### Chronic Pancreatitis

Chronic pancreatitis is an inflammatory condition whose symptoms can be similar to those associated with pancreatic cancer, and there are no good biomarkers to distinguish these two conditions [8]. Although it is uncommon for pancreatic cancer cases to be mistaken for pancreatitis, there is occasional difficulty in clinically distinguishing chronic pancreatitis from pancreatic cancer. Often, pancreatic cancer obstructs the pancreatic duct and causes an obstructive form of pancreatitis. Thus, biomarkers that can distinguish these two clinical conditions are desirable. This compendium has 372 molecules reported to be overexpressed in chronic pancreatitis. A partial list of molecules overexpressed in chronic pancreatitis is provided in Table E in Table S1. Many of the molecules overexpressed in chronic pancreatitis are also overexpressed in pancreatic cancer. For example, EPH receptor A3 and fibrillin 1 are overexpressed in the stromal compartment of both chronic pancreatitis and pancreatic cancer. Proteomic studies have been carried out to discover proteins that are differentially expressed between pancreatitis and pancreatic cancer [9]. Molecules that have been observed to be upregulated in chronic pancreatitis but not in PDACs include CCL3 and CCL4 [10]. On the other hand, molecules that are overexpressed in PDACs but not in chronic pancreatitis include annexin A2 and IGFBP2 [11]. Such molecules could be tested further, which might prove useful in distinguishing pancreatic cancer from chronic pancreatitis. Also, identifying biomarkers with better sensitivity and specificity than CA19-9 will significantly improve the diagnosis of pancreatic cancer. In fact, recent reports have shown that CEACAM1 and MUC1 indeed possess higher sensitivity and specificity for diagnosis of PDACs than CA19-9, the current gold standard [12], [13].

### Molecules Overexpressed in the Tumor Microenvironment

It is becoming clear that the tumor microenvironment is involved in initiation, proliferation, migration, invasion, angiogenesis, and metastasis of cancers. The role of the stromal component in pancreatic cancer progression is also being investigated, and recent reports provide experimental evidence to show the effect of the tumor microenvironment in promoting pancreatic cancers [14]. The significance of the tumor microenvironment is also exemplified in a study where expression of SPARC by peritumoral fibroblasts was shown to be associated with poorer prognosis for patients with pancreatic cancer [15]. During our literature search, in addition to information on the overexpression of molecules, we have also documented data on the particular cell types where they are overexpressed. There are more than 200 molecules in the compendium that have been reported to be overexpressed in pancreatic cancer-associated stroma; a subset of these have been listed in Table F in Table S1. We reasoned that a large number of molecules might be specifically overexpressed in the pancreatic cancer stroma because of the large stromal compartment in the pancreas and the frequent occurrence of an intense stromal reaction in PDACs. Surprisingly, as illustrated by the set of molecules listed in Table F in Table S1, most proteins that were overexpressed in the stroma of pancreatic cancer tissue were also overexpressed in the neoplastic ductal cells. Galectin 1 and CRISP-3 are among the minority of molecules that have been clearly described to be overexpressed only in the stroma, and not the neoplastic cells.

## Conclusions

Our efforts at creating this compendium represent the first step in tackling biomarkers for pancreatic cancer in a global and systematic fashion. In fact, it is already being used by a consortium of investigators who are developing antibodies against the 60 most promising targets in PDACs as part of a new initiative funded by the Lustgarten Foundation for Pancreatic Cancer Research. Our compendium also included data on other, less common subtypes of pancreatic cancer (Table G in Table S1 provides a partial list of molecules). The entire list of molecules overexpressed in pancreatic cancers that are included in the compendium is provided in Table S2. It must be pointed out that 74% of the molecules in this compendium are based solely on mRNA evidence. As is inherent to mRNA-based methods, especially DNA microarrays, the data often require subsequent validation by other methods. Further, several high-throughput studies carried out to identify genes that are differentially expressed in pancreatic cancer have used tissues that are not microdissected to separate cancer from stroma. Thus, it is unclear in many instances if the observed difference in the expression of a particular gene originates in the stroma or in cancer cells. This further underscores the importance of validating these observations using alternative methods by targeted studies.

Fortunately, about 648 molecules that we present have already been tested at the protein level (this includes 441 that have both mRNA and protein evidence) in individual laboratories, making them high-priority candidates for further testing. A significant number of candidate biomarkers are secreted and membrane-bound molecules, which makes them attractive candidates as biomarkers for early detection. The molecules described in this catalog can also serve as clinically relevant targets. For example, they could be targets for chemoprevention of pancreatic cancer in individuals with a strong family history of pancreatic cancer [16]. The recent demonstration that COX-2 is overexpressed in PanINs and in IPMNs suggests that COX-2 inhibitors might be a reasonable chemoprevention strategy [17]. The candidate biomarkers identified may also be useful as imaging targets. A number of the claudins have been shown to be overexpressed in pancreatic cancer and are potential markers for early detection of pancreatic cancer [18]. For example, iodine-125 radiolabeled anti-claudin-4 antibody has been employed for imaging pancreatic cancer using gamma scintigraphy and single-photon emission computed tomography/computed tomography [19]. Finally, the candidate biomarkers identified may be useful as therapeutic targets as well. In this regard, prostate stem cell antigen is a good example because although it is being tested as a therapeutic target for the treatment of prostate cancer, it is also overexpressed in pancreatic cancer [20].

## Challenges and Outlook

Given the explosion of data from multiple platforms, the information must be integrated before a systems view of cancers can emerge. In this regard, we have used pancreatic cancer as an example to create a resource that should serve as a model for other cancers. Discovering a single biomarker that would be both sensitive and specific for cancer of a given organ might be more difficult than discovering a panel of biomarkers. Identifying components to be used in such a panel would require systematic cataloging and testing of the most promising candidates that are available. To this end, we have carried out a systematic curation of the literature that took approximately over 7,000 person hours. This was an international effort and was possible because of concerted efforts of trained scientists at the Institute of Bioinformatics, where the majority of the curation work was carried out, working closely with several scientists in the United States. Such database efforts are crucial for systems biology approaches to human diseases because the data are not available in a single location and often not accessible to those without any bioinformatics experience. We feel that a multipronged approach to cancer will require not only continued discovery efforts, but also resources that maximize what we already know about cancers. Our future goal is to develop a Web-based searchable database of all molecular alterations in pancreatic cancer—from the genome to the proteome—that will help initiate a systems medicine approach to cancer.

## Supporting Information

Table S1 Tables A–G. Table A: Partial list of molecules overexpressed in the majority of PDACs. Table B: Partial list of secreted proteins that have been reported to be overexpressed in pancreatic cancers at mRNA and protein levels. Table C: Partial list of plasma membrane-bound proteins reported to be overexpressed in pancreatic cancers at mRNA and protein levels. Table D: Partial list of molecules overexpressed in precursor lesions. Table E: Partial list of molecules overexpressed in chronic pancreatitis, along with their expression status in PDAC. Table F: Partial list of molecules overexpressed in the stroma associated with pancreatic cancer. Table G: Partial list of molecules showing elevated expression in different subtypes of pancreatic cancer.
(4.52 MB DOC)

Table S2 List of molecules reported to be overexpressed in pancreatic cancers.
(1.95 MB XLS)

## Abbreviations

IPMN: intraductal papillary mucinous neoplasm
PanIN: pancreatic intraepithelial neoplasia
PDAC: pancreatic ductal adenocarcinoma
