# Supplementary material for: A Compendium of Potential Biomarkers of Pancreatic Cancer
Source: PLoS Med. 2009 Apr 7;6(4):e1000046. doi: 10.1371/journal.pmed.1000046 (PMC2661257; doi:10.1371/journal.pmed.1000046)
Supplement: Table S1 — Tables A–G. Table A: Partial list of molecules overexpressed in the majority of PDACs. Table B: Partial list of secreted proteins that have been reported to be overexpressed in pancreatic cancers at mRNA and protein levels. Table C: Partial list of plasma membrane-bound proteins reported to be overexpressed in pancreatic cancers at mRNA and protein levels. Table D: Partial list of molecules overexpressed in precursor lesions. Table E: Partial list of molecules overexpressed in chronic pancreatitis, along with their expression status in PDAC. Table F: Partial list of molecules overexpressed in the stroma associated with pancreatic cancer. Table G: Partial list of molecules showing elevated expression in different subtypes of pancreatic cancer. (4.52 MB DOC) [file pmed.1000046.s001.doc]

##### Table A

**Partial list of molecules overexpressed in majority of pancreatic ductal adenocarcinomas**

|  | **Gene symbol** | **Protein name** | **Whether detectable in body fluids/plasma membrane** | **Invasive ductal adenocarcinoma** | | **Chronic Pancreatitis** | |
| --- | --- | --- | --- | --- | --- | --- | --- |
|  |  |  |  | **Method** | **Reference** | **Method** | **Reference** |
| **1.** | **MUC1** | **Mucin1** | **Pancreatic juice:** Gronborg, M et al., 2004  **Milk:** Muller, S et al., 1997  **Serum:** Storr, SJ et al., 2008, Moreno, M et al., 2007, Gold, DV et al., 2006  **Plasma membrane:** Wykes, M et al., 2002, Tsutsumida, H et al., 2006, Raina, D et al., 2006, Li, Y et al., 2001, Tajiri, T et al., 2004, Qu, CF et al., 2004 | **IHC** | Chhieng, DC et al., 2003 | **NA** | |
|  |  |  |  | **IHC** | Tajiri, T et al., 2004 |  |  |
|  |  |  |  | **IHC** | Luttges, J et al., 2001 |  |  |
|  |  |  |  | **IHC** | Qu, CF et al., 2004 |  |  |
|  |  |  |  | **RT-PCR, IHC** | Ohuchida, K et al., 2006 |  |  |
|  |  |  |  | **IHC** | Saitou, M et al., 2005 |  |  |
|  |  |  |  | **RT-PCR** | Andrianifahanana, M et al., 2001 |  |  |
|  |  |  |  | **SAGE** | Hustinx, SR et al., 2004 |  |  |
| **2.** | **MMP7** | **Matrix metallopeptidase 7 (matrilysin, uterine)** | **Seminal plasma:** Pilch, B et al., 2006  **Serum:** Maurel, J et al., 2007, Laszlo, A et al., 1990  **Plasma membrane:** Nishikawa, N et al., 2006 | **DNA Microarray** | Iacobuzio-Donahue, CA et al., 2003 | **NA** | |
|  |  |  |  | **DNA Microarray** | Laurell, H et al., 2006 |  |  |
|  |  |  |  | **DNA Microarray** | Crnogorac-Jurcevic, T et al., 2002 |  |  |
|  |  |  |  | **SAGE** | Hustinx, SR, et al., 2004 |  |  |
|  |  |  |  | **DNA Microarray** | Iacobuzio-Donahue, CA et al., 2002 |  |  |
|  |  |  |  | **Northern blot, ISH** | Bramhall, SR et al., 1997 |  |  |
|  |  |  |  | **IHC** | Nishikawa, N, et al., 2006 |  |  |
|  |  |  |  | **IHC** | Li, YJ et al., 2005 |  |  |
| **3.** | **ANXA2** | **Annexin A2** | **Serum:** Sheng, S et al., 2006  **Seminal Plasma:** Pilch, B, et al., 2006  **Tear:** de Souza, GA et al., 2006  **Saliva:** Neyraud, E et al., 2006  **Plasma membrane**: Wang, W et al., 2002, Barwise, JL et al., 1996, Tian, R et al., 2008, Paciucci, R et al., 1998, Esposito, I et al., 2006 | **DNA Microarray, IHC** | Esposito, I, et al., 2006 | **NA** | |
|  |  |  |  | **IHC** | Esposito, I, et al., 2006 |  |  |
|  |  |  |  | **Western Blot, IHC** | Chen, R, et al., 2007 |  |  |
|  |  |  |  | **Western Blot, IHC, ICAT** | Chen, R et al., 2005 |  |  |
|  |  |  |  | **2D Gel, MS, Western Blot, IHC** | Tian, R, et al., 2008 |  |  |
|  |  |  |  | **Western Blot** | Crnogorac-Jurcevic, T et al., 2005 |  |  |
|  |  |  |  | **IHC** | Ortiz-Zapater, E et al., 2007 |  |  |
|  |  |  |  | **IHC** | Paciucci, R, et al., 1998 |  |  |
| **4.** | **BIRC5** | **Baculoviral IAP repeat-containing 5 (survivin)** |  | **DNA Microarray** | Nakamura, T et al., 2004 |  | |
|  |  |  |  | **RT-PCR** | Lopes, RB et al., 2007 |  |  |
|  |  |  |  | **RT-PCR, Western Blot** | Satoh, K et al., 2001 |  |  |
|  |  |  |  | **RT-PCR, ISH, IHC** | Lopes, RB, et al., 2007 |  |  |
|  |  |  |  | **IHC** | Wei, H et al., 2006 |  |  |
|  |  |  |  | **IHC** | Qiao, JG et al., 2004 |  |  |
|  |  |  |  | **IHC** | Bhanot, U et al., 2006 |  |  |
|  |  |  |  | **IHC** | Sun, HC et al., 2007 |  |  |
| **5.** | **CASP3** | **Caspase 3, apoptosis-related cysteine peptidase** |  | **RT-PCR, IHC** | Satoh, K et al., 2000 |  | |
|  |  |  |  | **RT-PCR, IHC** | Carbone, A et al., 2005 |  |  |
|  |  |  |  | **IHC** | Virkajarvi, N et al., 1998 |  |  |
|  |  |  |  | **IHC** | Meggiato, T et al., 2003 |  |  |
| **6.** | **CENPF** | **Centromere protein F (mitosin)** |  | **DNA Microarray, IHC** | Grutzmann, R et al., 2004 |  | |
|  |  |  |  | **IHC** | Grutzmann, R et al., 2005 |  |  |
| **7.** | **PTGS2** | **Prostaglandin-endoperoxide synthase 2 (prostaglandin G/H synthase and cyclooxygenase)** | **Seminal Plasma:** States, DJ et al., 2006 | **RT-PCR, IHC** | Tucker, ON et al., 1999 | **Western Blot, IHC** | Schlosser, W et al., 2002 |
|  |  |  |  | **RT-PCR, Western Blot, IHC** | Okami, J et al., 1999 |  |  |
|  |  |  |  | **IHC** | Albazaz, R et al., 2005 |  |  |
|  |  |  |  | **IHC** | Wang, HX et al., 2003 |  |  |
|  |  |  |  | **Western Blot** | Franco, L et al., 2004 |  |  |
| **8.** | **SPP1** | **Secreted phosphoprotein 1 (osteopontin, bone sialoprotein I, early T-lymphocyte activation 1)** | **Serum:** Kang, SY et al., 2007, Koopmann, J et al., 2006  **Hemodialysis Fluid**: Molina, H et al., 2005  **Urine**: Christensen, B et al., 2008, Adachi, J et al., 2006  **Cerebrospinal Fluid**: Zougman, A et al., 2008 | **DNA Microarray** | Rogers, CD et al., 2006 | **DNA Microarray** | Fukushima, N et al., 2005 |
|  |  |  |  | **DNA Microarray** | Jin, G et al., 2005 | **IHC, ELISA, RT-PCR** | Kolb, A et al., 2005 |
|  |  |  |  | **DNA Microarray** | Fukushima, N, et al., 2005 | **NA** | |
|  |  |  |  | **IHC, ELISA** | Koopmann, J et al., 2004 |  |  |
|  |  |  |  | **IHC, ELISA, RT-PCR** | Kolb, A, et al., 2005 |  |  |
|  |  |  |  | **IHC** | Coppola, D et al., 2004 |  |  |
| **9.** | **FSCN1** | **Fascin homolog 1, actin-bundling protein (Strongylocentrotus purpuratus)** | **Blood**: Sonderbye, L et al., 1998  **Plasma membrane:** Lin, XH et al., 1996, Tsai, WC et al., 2007, Tsai, WC et al., 2007 | **DNA Microarray** | Iacobuzio-Donahue, CA, et al., 2003 | **NA** | |
|  |  |  |  | **DNA Microarray** | Logsdon, CD et al., 2003 |  |  |
|  |  |  |  | **DNA Microarray** | Nakamura, T, et al., 2004 |  |  |
|  |  |  |  | **DNA Microarray** | Iacobuzio-Donahue, CA, et al., 2002 |  |  |
|  |  |  |  | **ICAT** | Chen, R, et al., 2005 |  |  |
|  |  |  |  | **2D Gel, IHC** | Lu, Z et al., 2004 |  |  |
|  |  |  |  | **Western Blot** | Crnogorac-Jurcevic, T, et al., 2005 |  |  |
|  |  |  |  | **IHC** | Iacobuzio-Donahue, CA, et al., 2002 |  |  |
| **10.** | **S100A6** | **S100 calcium binding protein A6** | **Hemodialysis Fluid:** Molina, H, et al., 2005  **Tear:** de Souza, GA, et al., 2006 | **DNA Microarray, IHC** | Logsdon, CD, et al., 2003 | **NA** | |
|  |  |  |  | **DNA Microarray, IHC** | Crnogorac-Jurcevic, T et al., 2003 |  |  |
|  |  |  |  | **DNA Microarray** | Buchholz, M et al., 2005 |  |  |
|  |  |  |  | **2D Gel, IHC** | Shekouh, AR et al., 2003 |  |  |
|  |  |  |  | **IHC** | Ohuchida, K et al., 2005 |  |  |
| **11.** | **SERPINB5** | **Serpin peptidase inhibitor, clade B (ovalbumin), member 5** | **Seminal Plasma:** Pilch, B, et al., 2006  **Tear:** de Souza, GA, et al., 2006  **Plasma membrane:** Sager, R et al., 1997 | **DNA Microarray** | Crnogorac-Jurcevic, T, et al., 2003 | **NA** | |
|  |  |  |  | **DNA Microarray** | Pfeffer, F et al., 2004 |  |  |
|  |  |  |  | **IHC** | Maass, N et al., 2001 |  |  |
|  |  |  |  | **IHC** | Cao, D et al., 2007 |  |  |
|  |  |  |  | **IHC** | Lim, YJ et al., 2004 |  |  |
|  |  |  |  | **IHC** | Ohike, N et al., 2003 |  |  |

**Table B**

**A partial list of secreted proteins that have been reported to be overexpressed in pancreatic cancers at mRNA and protein level**

|  | **Gene Symbol** | **Protein name** | **Whether detectable in body fluids** | **Evidence of overexpression at**  **mRNA level** | **Evidence of overexpression at**  **protein level** |
| --- | --- | --- | --- | --- | --- |
| **1.** | **MUC2** | **Mucin 2** | **Bile:** Kristiansen, TZ et al., 2004  **Pancreatic juice:** Hibi, Y et al., 2007  **Airway Mucus:** Hovenberg, HW et al., 1996 | **qRT-PCR:** Andrianifahanana, M, et al., 2001, | **Immunohistochemistry:** Luttges, J et al., 2002, Maitra, A et al., 2003, Hanski, C et al., 1997 |
| **2.** | **SPP1** | **Osteopontin** | **Serum:** Koopmann, J, et al., 2004, Kolb, A, et al., 2005, Kang, SY, et al., 2007, Koopmann, J, et al., 2006, Molina, H, et al., 2005  **Urine:** Christensen, B et al., 2007 , Adachi, J, et al., 2006 | **DNA microarray:** Missiaglia, E et al., 2004, Rogers, CD, et al., 2006, Jin, G, et al., 2005, Fukushima, N, et al., 2005  **qRT-PCR:** Kolb, A, et al., 2005 | **Immunohistochemistry:** Koopmann, J, et al., 2004, Coppola, D, et al., 2004, Kolb, A, et al., 2005  **ELISA:** Koopmann, J, et al., 2004, Kolb, A, et al., 2005 |
| **3.** | **SPINK1** | **Serine protease inhibitor** | **Serum:** Molina, H, et al., 2005  **Pancreatic juice:** Chen, R et al., 2006  **Urine:** Huhtala, ML et al., 1982, Adachi, J, et al., 2006  **Gastric juice:** Freeman, TC et al., 1990 | **SAGE:** Hustinx, SR, et al., 2004 | **ICAT:** Chen, R et al., 2007, Chen, R, et al., 2006 |
| **4.** | **MSLN** | **Mesothelin** | **Serum:** Cristaudo, A et al., 2007, Creaney, J et al., 2007, Hassan, R et al., 2006, Onda, M et al., 2006  **Urine:** Badgwell, D et al., 2007, Adachi, J, et al., 2006 | **DNA microarray:** Iacobuzio-Donahue, CA, et al., 2003, Logsdon, CD, et al., 2003, Sato, N et al., 2004  **SAGE:** Argani, P et al., 2001, Hustinx, SR, et al., 2004  **qRT-PCR:** Argani, P, et al., 2001, Watanabe, H et al., 2005, Sato, N, et al., 2004 | **Immunohistochemistry:** Hassan, R et al., 2005, Watanabe, H, et al., 2005, Lohr, M et al., 1996, Maitra, A, et al., 2003, Argani, P, et al., 2001, Sato, N, et al., 2004, Ordonez, NG, 2003 |
| **5.** | **ADM** | **Adrenomedullin** | **Serum:** Keleg, S et al., 2007  **Plasma:** Nakatsuka, M et al., 2003, Kitamura, K et al., 1994  **Saliva:** Kapas, S et al., 2004  Urine: Adachi, J, et al., 2006 | **DNA microarray:** Nakamura, T, et al., 2004  **qRT-PCR:** Ramachandran, V et al., 2007, Keleg, S, et al., 2007 | **Immunohistochemistry:** Ramachandran, V, et al., 2007, Pavel, ME et al., 2006, Ishikawa, T et al., 2003, Keleg, S, et al., 2007 |
| **6.** | **TFF1** | **Trefoil factor 1** | **Serum:** Gronbaek, H et al., 2006, Miyashita, S et al., 1994 Vestergaard, EM et al., 2004  **Urine:** Miyashita, S, et al., 1994, Chenard, MP et al., 2004, Adachi, J, et al., 2006 | **DNA microarray:** Fukushima, N et al., 2004, Johnson, SK et al., 2006, Terris, B et al., 2002, Friess, H et al., 2003, Buchholz, M, et al., 2005, Prasad, NB et al., 2005  **qRT-PCR:** Johnson, SK, et al., 2006, Prasad, NB, et al., 2005  **SAGE:** Terris, B, et al., 2002 | **Immunohistochemistry:** Terris, B, et al., 2002, Prasad, NB, et al., 2005 |
| **7.** | **LCN2** | **Lipocalin 2** | **Plasma:** Axelsson, L et al., 1995, Blaser, J et al., 1995  **Serum:** Molina, H, et al., 2005  **Bile:** Kristiansen, TZ, et al., 2004  **Pancreatic juice:** Gronborg, M, et al., 2004  **Urine:** Blaser, J, et al., 1995, Adachi, J, et al., 2006  **Synovial fluids:** Blaser, J, et al., 1995 | **DNA microarray:** Terris, B, et al., 2002, Iacobuzio-Donahue, CA, et al., 2003, Iacobuzio-Donahue, CA et al., 2003, Laurell, H, et al., 2006, Iacobuzio-Donahue, CA, et al., 2002  **qRT-PCR:** Laurell, H, et al., 2006  **SAGE:** Terris, B, et al., 2002, Argani, P et al., 2001, Hustinx, SR, et al., 2004 | **Immunohistochemistry:** Laurell, H, et al., 2006, Terris, B, et al., 2002 |
| **8.** | **POSTN** | **Periostin** | **Serum:** Sasaki, H et al., 2003, Sasaki, H et al., 2002, Sasaki, H et al., 2001, Baril, P et al., 2007 | **DNA microarray:** Crnogorac-Jurcevic, T, et al., 2003, Johnson, SK, et al., 2006, Iacobuzio-Donahue, CA, et al., 2002, Friess, H, et al., 2003  **qRT-PCR:** Erkan, M et al., 2007, Friess, H, et al., 2003  **In situ hybridization:** Friess, H, et al., 2003 | **ELISA:** Baril, P, et al., 2007  **ICAT:** Chen, R, et al., 2005  **Western blotting:** Erkan, M, et al., 2007 |
| **9.** | **TIMP1** | **Tissue inhibitor of metalloproteinase 1** | **Serum:** Zhou, W et al., 1998,  **Plasma:** Yukawa, N et al., 2007, Sorensen, NM et al., 2007, Caspersen, MB et al., 2007  **Gingival crevicular fluid**: Emingil, G et al., 2006  **Urine:** Adachi, J, et al., 2006 | **DNA microarray:** Iacobuzio-Donahue, CA, et al., 2002, Iacobuzio-Donahue, CA, et al., 2003, Iacobuzio-Donahue, CA, et al., 2003, Jin, G, et al., 2005, Laurell, H, et al., 2006, Sato, N, et al., 2004  **SAGE:** Crnogorac-Jurcevic, T, et al., 2002  **Northern blot:** Zhou, W, et al., 1998, Bramhall, SR, et al., 1997 | **Immunohistochemistry:** Sato, N, et al., 2004  **ELISA:** Zhou, W, et al., 1998 |
| **10.** | **MMP11** | **Matrix metalloproteinase 11** | **Serum:** Yang, YH et al., 2008, Tirumalai, RS et al., 2003 | **DNA microarray:** Nakamura, T, et al., 2004, Crnogorac-Jurcevic, T, et al., 2003, Iacobuzio-Donahue, CA, et al., 2003, Grutzmann, R, et al., 2004, Laurell, H, et al., 2006, Grutzmann, R, et al., 2004  **SAGE:** Hustinx, SR, et al., 2004  **Northern blot:** von Marschall, Z et al., 1998, Bramhall, SR, et al., 1997 | **Immunohistochemistry:** Grutzmann, R, et al., 2005, von Marschall, Z, et al., 1998  **Western blotting:** von Marschall, Z, et al., 1998 |
| **11.** | **REG3A** | **Regenerating islet derived 3 alpha** | **Pancreatic juice:** Rosty, C et al., 2002, Chen, R, et al., 2007, Chen, R, et al., 2006, Gronborg, M, et al., 2004, Motoo, Y et al., 2001  **Serum:** Molina, H, et al., 2005 | **DNA microarray:** Fukushima, N, et al., 2005,  **qRT-PCR:** Fukushima, N, et al., 2005, Rosty, C, et al., 2002 | **Immunohistochemistry:** Xie, MJ et al., 2003, Fukushima, N, et al., 2005, Rosty, C, et al., 2002  **ICAT:** Chen, R, et al., 2007, Chen, R, et al., 2006  **ELISA:** Cerwenka, H et al., 2001 |
| **12.** | **MMP9** | **Matrix metalloproteinase 9** | **Serum:** Hlatky, MA et al., 2007, Guo, CB et al., 2007, Wilson, S et al., 2006  **Tear:** de Souza, GA, et al., 2006  **Urine:** Adachi, J, et al., 2006 | **DNA microarray:** Nakamura, T, et al., 2004, Segara, D et al., 2005, Friess, H, et al., 2003  **Northern blot:** Wagner, M et al., 1999, Kleeff, J et al., 1999  **qRT-PCR:** Grutzmann, R, et al., 2005 | **Immunohistochemistry:** Gurevich, LE, 2003, Harvey, SR et al., 2003, Qian, X et al., 2001 |
| **13.** | **SPARCL1** | **Spark like 1** | **Serum:** Tirumalai, RS, et al., 2003  **CSF:** Scalabrini, D et al., 2007  **Urine:** Adachi, J, et al., 2006 | **DNA microarray:** Esposito, I et al., 2007, Kayton, ML et al., 2003  **qRT-PCR:** Esposito, I, et al., 2007  **SAGE:** Ryu, B et al., 2001 | **Immunohistochemistry:** Esposito, I, et al., 2007 |
| **14.** | **MUC5AC** | **Mucin 5AC** | **Serum:** Bamrungphon, W et al., 2007, Boonla, C et al., 2003, Kocer, B et al., 2006 | **DNA microarray:** Iacobuzio-Donahue, CA, et al., 2003, Sato, N, et al., 2004, Grutzmann, R et al., 2003  **qRT-PCR:** Ohuchida, K, et al., 2006 | **Immunohistochemistry:** Iacobuzio-Donahue, CA, et al., 2003, Kanno, A et al., 2006, Luttges, J, et al., 2002, Ohuchida, K, et al., 2006 |

**Table C**

**A partial list of plasma membrane bound proteins reported to be overexpressed in pancreatic cancers at mRNA and protein level**

|  | **Gene Symbol** | **Protein name** | **Membrane bound**  **(Literature)** | **Whether detectable in body fluids** | **Evidence of overexpression at**  **mRNA level** | **Evidence of overexpression at**  **protein level** |
| --- | --- | --- | --- | --- | --- | --- |
| **1** | **MUC4** | **Mucin-4** | Ramsauer, VP et al., 2003, Duraisamy, S et al., 2006, Alameda, F et al., 2007, Zhang, J et al., 2006 | **Secreted:** Moniaux, N et al., 2000, Choudhury, A et al., 2000  **Tear:** Spurr-Michaud, S et al., 2007  **Saliva:** Liu, B et al., 1998 | **DNA microarray:** Iacobuzio-Donahue, CA, et al., 2003, Segara, D, et al., 2005  **qRT-PCR:** Singh, AP et al., 2007, Li, XH et al., 2005, Choudhury, A et al., 2004, Andrianifahanana, M, et al., 2001  **Northern blotting:** Singh, AP, et al., 2007, Choudhury, A, et al., 2004  **In situ hybridization:** Park, HU et al., 2003 | **Western blotting:**  Singh, AP, et al., 2007, Moniaux, N et al., 2004 **Immunohistochemistry:** Kanno, A, et al., 2006, Bhardwaj, A et al., 2007, Park, HU, et al., 2003, Singh, AP, et al., 2007, Moniaux, N, et al., 2004, Saitou, M, et al., 2005, Swartz, MJ et al., 2002 |
| **2** | **ITGA6** | **Integrin alpha 6** | Cruz-Monserrate, Z et al., 2007 |  | **DNA microarray:** Sato, N, et al., 2004 | **Western blotting:** Sawai, H et al., 2006,  **Immunohistochemistry:** Cruz-Monserrate, Z, et al., 2007, Gesierich, S et al., 2005, Halatsch, ME et al., 1997, Sawai, H, et al., 2006 |
| **3** | **PLAUR** | **Plasminogen activator, urokinase receptor** | Min, HY et al., 1992, Mukhina, S et al., 2000, Casey, JR et al., 1994, Shin, BK et al., 2003, Paciucci, R, et al., 1998 | **Plasma:** Kasperska-Zajac, A et al., 2007, Grebenchtchikov, N et al., 2005, Florquin, S et al., 2001, Shariat, SF et al., 2007  **Urine:** Florquin, S, et al., 2001  **Cerebrospinal fluid:** Sporer, B et al., 2005 | **DNA microarray:** Iacobuzio-Donahue, CA, et al., 2002, Iacobuzio-Donahue, CA, et al., 2003, Iacobuzio-Donahue, CA, et al., 2003, Logsdon, CD, et al., 2003, Segara, D, et al., 2005, Jin, G, et al., 2005  **Northern blotting:**  Lohr, M, et al., 1996 | **Western blotting:** Sawai, H, et al., 2006, Paciucci, R, et al., 1998, Lohr, M, et al., 1996  **Immunohistochemistry:** Sawai, H, et al., 2006, Paciucci, R, et al., 1998 |
| **4** | **FXYD3** | **FXYD domain-containing ion transport regulator 3** | Arimochi, J et al., 2007, Kayed, H et al., 2006, Morrison, BW et al., 1995 |  | **DNA microarray:**  Iacobuzio-Donahue, CA, et al., 2003, Iacobuzio-Donahue, CA, et al., 2003, Friess, H, et al., 2003, Logsdon, CD, et al., 2003, Kayed, H, et al., 2006, Sato, N, et al., 2004  **Northern blot:** Friess, H, et al., 2003  **qRT-PCR:**  Kayed, H, et al., 2006 | **Immunohistochemistry:**  Kayed, H, et al., 2006 |
| **5** | **ADAM9** | **A disintegrin and metalloproteinase domain 9** | Weskamp, G et al., 1996, Grutzmann, R et al., 2004 | **Urine: Adachi, J, et al., 2006** | **DNA microarray:**  Iacobuzio-Donahue, CA, et al., 2003, Grutzmann, R, et al., 2003  **qRT-PCR:**  Grutzmann, R, et al., 2005 | **Immunohistochemistry:**  Grutzmann, R, et al., 2004, Besleaga, R et al., 2003 |
| **6** | **BSG** | **Basigin** | Berditchevski, F et al., 1997, Zhang, W et al., 2007 | **Serum:** Zhang, W, et al., 2007  **Gingival crevicular fluid:** Emingil, G, et al., 2006 | **qRT-PCR:**  Li, M et al., 2006, Zhang, W, et al., 2007 | **Western blotting:** Li, M, et al., 2006, Zhang, W, et al., 2007  **Immunohistochemistry:**  Schneiderhan, W et al., 2007, Li, M, et al., 2006, Riethdorf, S et al., 2006, Zhang, W, et al., 2007  **ELISA:** Zhang, W, et al., 2007  **ICAT:** Chen, R, et al., 2005 |
| **7** | **CEACAM5** | **Carcinoembryonic antigen related cell adhesion molecule 5** | Guignot, J et al., 2000, Screaton, RA et al., 2000 | **Plasma:** Anderson, NL et al., 2004  **Pancreatic juice:** Gronborg, M, et al., 2004 | **DNA microarray:**  Sato, N, et al., 2004, Blumenthal, RD et al., 2007, Buchholz, M, et al., 2005, Iacobuzio-Donahue, CA, et al., 2003, Iacobuzio-Donahue, CA, et al., 2003, Logsdon, CD, et al., 2003, Johnson, SK, et al., 2006, Kristiansen, G et al., 2006, Friess, H, et al., 2003 | **Immunohistochemistry:**  Blumenthal, RD, et al., 2007, Buchholz, M, et al., 2005,  **Mass spectrometry:** Gronborg, M, et al., 2004 |
| **8** | **CXCR4** | **Chemokine, CXC motif, receptor 4** | Ding, Z et al., 2003, Babcock, GJ et al., 2003, Babcock, GJ, et al., 2003 |  | **DNA microarray:** Rogers, CD, et al., 2006, Sato, N, et al., 2004, Johnson, SK, et al., 2006, Maitra, A et al., 2003 | **Immunohistochemistry:** Sato, N, et al., 2004 |
| **9** | **NRP1** | **Neuropilin 1** | Man, XY et al., 2006, Ghez, D et al., 2006 | **Plasma:** States, DJ, et al., 2006  **Secreted:** Gagnon, ML et al., 2000 | **Northern blotting:** Parikh, AA et al., 2003  **qRT-PCR:** Fukahi, K et al., 2004, Muller, MW et al., 2007 | **Western blotting:** Hansel, DE et al., 2004  **Immunohistochemistry:** Parikh, AA, et al., 2003, Fukahi, K, et al., 2004, Muller, MW, et al., 2007, Hansel, DE, et al., 2004 |
| **10** | **JAG1** | **Jagged 1** | Morrissette, JD et al., 2001 | **Secreted:** Aho, S, 2004 | **DNA microarray:** Fukushima, N, et al., 2004, Prasad, NB, et al., 2005  qRT-PCR: Buchler, P et al., 2005 | **Immunohistochemistry:** Fukushima, N, et al., 2004, Buchler, P, et al., 2005 |
| **11** | **CLDN4** | **Claudin 4** | Ueda, J et al., 2007, Nichols, LS et al., 2004 |  | **DNA microarray:** Sato, N, et al., 2004, Buchholz, M, et al., 2005, Iacobuzio-Donahue, CA, et al., 2003, Iacobuzio-Donahue, CA, et al., 2003, Terris, B, et al., 2002  **qRT-PCR:** Iacobuzio-Donahue, CA, et al., 2003  **SAGE:** Terris, B, et al., 2002  **Northern blot:** Michl, P et al., 2001 | **Immunohistochemistry:** Nichols, LS, et al., 2004, Sato, N, et al., 2004  **Immunolabeling:** Foss, CA et al., 2007 |

##### Table D

##### Partial list of molecules overexpressed in precursor lesions

|  | **Gene Symbol** | **Protein name** | **Whether detectable in body fluids/plasma membrane** | **PanIN** | | **IPMN** | | **PDAC** | |
| --- | --- | --- | --- | --- | --- | --- | --- | --- | --- |
|  |  |  |  | **Method** | **Reference** | **Method** | **Reference** | **Method** | **Reference** |
| **1.** | **ANXA2** | **Annexin A2** | **Serum:** Sheng, S, et al., 2006  **Saliva:** Neyraud, E, et al., 2006  **Seminal Plasma:** Pilch, B, et al., 2006  **Tear:** de Souza, GA, et al., 2006  **Plasma membrane:** Barwise, JL, et al., 1996, Wang, W, et al., 2002, Tian, R, et al., 2008, Paciucci, R, et al., 1998, Esposito, I, et al., 2006 | **IHC** | Ortiz-Zapater, E, et al., 2007 | **NA** |  | **Western Blot, IHC** | Chen, R, et al., 2007 |
|  |  |  |  | **IHC** | Esposito, I, et al., 2006 |  |  | **Western Blot, IHC, ICAT** | Chen, R, et al., 2005 |
|  |  |  |  | **DIGE** | Sitek, B et al., 2005 |  |  | **2D gel, Western Blot, MS, IHC** | Tian, R, et al., 2008 |
|  |  |  |  |  |  |  |  | **Western Blot** | Crnogorac-Jurcevic, T, et al., 2005 |
|  |  |  |  |  |  |  |  | **IHC** | Ortiz-Zapater, E, et al., 2007 |
|  |  |  |  |  |  |  |  | **IHC** | Paciucci, R, et al., 1998 |
|  |  |  |  |  |  |  |  | **DNA Microarray,IHC** | Esposito, I, et al., 2006 |
| **2.** | **CD55** | **CD55 molecule, decay accelerating factor for complement (Cromer blood group)** | **Bile:** Kristiansen, TZ, et al., 2004  **Plasma membrane:** Hindmarsh, EJ et al., 1998, Taylor, CT et al., 1996 | **NA** |  | **DNA Microarray** | Terris, B, et al., 2002 | **DNA Microarray** | Iacobuzio-Donahue, CA, et al., 2003 |
|  |  |  |  |  |  |  |  | **DNA Microarray** | Friess, H, et al., 2003 |
|  |  |  |  |  |  | **DNA Microarray, IHC** | Sato, N, et al., 2004 | **DNA Microarray** | Logsdon, CD, et al., 2003 |
|  |  |  |  |  |  |  |  | **DNA Microarray** | Lowe, AW et al., 2007 |
|  |  |  |  |  |  |  |  | **DNA Microarray, RT-PCR** | Johnson, SK, et al., 2006 |
| **3.** | **CEACAM6** | **Carcinoembryonic antigen-related cell adhesion molecule 6 (non-specific cross reacting antigen)** | **Pancreatic Juice:** Gronborg, M, et al., 2004  **Urine:** Adachi, J, et al., 2006  **Saliva:** Ramachandran, P et al., 2006  **Plasma membrane:** Kuroki, M et al., 1988, Barnett, T et al., 1988, Duxbury, MS et al., 2005 | **IHC** | Duxbury, MS, et al., 2005 | **NA** |  | **IHC** | Blumenthal, RD, et al., 2007 |
|  |  |  |  |  |  |  |  | **DNA Microarray** | Johnson, SK, et al., 2006 |
|  |  |  |  |  |  |  |  | **DNA Microarray** | Logsdon, CD, et al., 2003 |
|  |  |  |  |  |  |  |  | **SAGE** | Hustinx, SR, et al., 2004 |
|  |  |  |  |  |  |  |  | **IHC** | Duxbury, MS, et al., 2005 |
|  |  |  |  |  |  |  |  | **DNA Microarray** | Friess, H, et al., 2003 |
|  |  |  |  |  |  |  |  | **RT-PCR, Northern Blot** | Gress, TM et al., 1997 |
|  |  |  |  |  |  |  |  | **DNA Microarray** | Iacobuzio-Donahue, CA, et al., 2003 |
|  |  |  |  |  |  |  |  | **SAGE** | Ryu, B et al., 2002 |
| **4.** | **CLDN4** | **Claudin 4** | **Plasma membrane:** Nichols, LS, et al., 2004, Katahira, J et al., 1997 | **IHC** | Nichols, LS, et al., 2004 | **DNA Microarray** | Terris, B, et al., 2002 | **Northern Blot, IHC** | Michl, P, et al., 2001 |
|  |  |  |  |  |  | **DNA Microarray,**  **IHC** | Sato, N, et al., 2004 | **DNA Microarray** | Lowe, AW, et al., 2007 |
|  |  |  |  |  |  |  |  | **DNA Microarray** | Buchholz, M, et al., 2005 |
|  |  |  |  |  |  |  |  | **DNA Microarray** | Iacobuzio-Donahue, CA, et al., 2003 |
|  |  |  |  |  |  |  |  | **SAGE** | Terris, B, et al., 2002 |
|  |  |  |  |  |  |  |  | **IHC** | Nichols, LS, et al., 2004 |
|  |  |  |  |  |  |  |  | **SAGE** | Ryu, B, et al., 2002 |
| **5.** | **CTSE** | **Cathepsin E** | **Pancreatic juice:** Azuma, T et al., 1996, Uno, K et al., 2000  **Plasma membrane:** Takeda-Ezaki, M et al., 1993 | **DNA Microarary** | Prasad, NB, et al., 2005 | **DNA Microarray** | Terris, B, et al., 2002 | **DNA Microarray** | Fukushima, N, et al., 2004 |
|  |  |  |  |  |  | **DNA Microarray** | Sato, N, et al., 2004 | **DNA Microarray** | Lowe, AW, et al., 2007 |
|  |  |  |  |  |  |  |  | **DNA Microarray, IHC** | Buchholz, M, et al., 2005 |
|  |  |  |  |  |  |  |  | **DNA Microarray** | Iacobuzio-Donahue, CA, et al., 2003 |
|  |  |  |  |  |  |  |  | **SAGE** | Terris, B, et al., 2002 |
|  |  |  |  |  |  |  |  | **DNA Microarray** | Johnson, SK, et al., 2006 |
|  |  |  |  |  |  |  |  | **DNA Microarray** | Friess, H, et al., 2003 |
|  |  |  |  |  |  |  |  | **DNA Microarray** | Pfeffer, F, et al., 2004 |
| **6.** | **MUC5AC** | **Mucin 5AC, oligomeric mucus/gel-forming** | **Serum:** Bamrungphon, W, et al., 2007, Boonla, C, et al., 2003  **Pancreatic juice:** Chen, R, et al., 2007  **Tear:** de Souza, GA, et al., 2006 | **IHC** | Maitra, A, et al., 2003 | **IHC** | Kanno, A, et al., 2006 | **DNA Microarray, IHC** | Iacobuzio-Donahue, CA, et al., 2003 |
|  |  |  |  |  |  |  |  | **DNA Microarray** | Lowe, AW, et al., 2007 |
|  |  |  |  |  |  |  |  | **RT-PCR, IHC** | Ohuchida, K, et al., 2006 |
|  |  |  |  |  |  |  |  | **DNA Microarray** | Grutzmann, R, et al., 2003 |
|  |  |  |  |  |  |  |  | **DNA Microarray** | Friess, H, et al., 2003 |
|  |  |  |  |  |  | **DNA Microarray** | Sato, N, et al., 2004 | **IHC** | Luttges, J, et al., 2002 |
| **7.** | **NQO1** | **NAD(P)H dehydrogenase, quinone 1** | **Tear:** de Souza, GA, et al., 2006  **Plasma membrane:** Forthoffer, N et al., 2002 | **IHC** | Lewis, AM et al., 2005 | **NA** |  | **DNA Microarray** | Nakamura, T, et al., 2004 |
|  |  |  |  | **DNA Microarray** | Prasad, NB, et al., 2005 |  |  | **DNA Microarray** | Lowe, AW, et al., 2007 |
|  |  |  |  |  |  |  |  | **IHC, Other assays** | Lewis, AM, et al., 2005 |
|  |  |  |  |  |  |  |  | **DNA Microarray** | Iacobuzio-Donahue, CA, et al., 2003 |
|  |  |  |  |  |  |  |  | **DNA Microarray** | Iacobuzio-Donahue, CA, et al., 2003 |
|  |  |  |  |  |  |  |  | **DNA Microarray** | Logsdon, CD, et al., 2003 |
|  |  |  |  |  |  |  |  | **DNA Microarray** | Grutzmann, R, et al., 2004 |
| **8.** | **LGALS3** | **Lectin, galactoside-binding, soluble, 3** | **Hemodialysis fluid:** Molina, H, et al., 2005  **Seminal plasma:** Pilch, B, et al., 2006  **Tear:** de Souza, GA, et al., 2006 | **NA** |  | **DNA Microarray, IHC** | Terris, B, et al., 2002 | **SAGE, IHC** | Terris, B, et al., 2002 |
|  |  |  |  |  |  |  |  | **DNA Microarray** | Lowe, AW, et al., 2007 |
|  |  |  |  |  |  |  |  | **DNA Microarry** | Iacobuzio-Donahue, CA, et al., 2003 |
|  |  |  |  |  |  |  |  | **DNA Microarray, RT-PCR** | Johnson, SK, et al., 2006 |
|  |  |  |  |  |  |  |  | **Northern Blot, ISH, Western Blot, IHC** | Berberat, PO et al., 2001 |
|  |  |  |  |  |  |  |  | **Western Blot** | Shen, J et al., 2004 |
| **9.** | **CLDN18** | **Claudin 18** | **Plasma membrane:** Niimi, T et al., 2001, Karanjawala, ZE et al., 2008 | **IHC** | Karanjawala, ZE, et al., 2008 | **DNA Microarray** | Sato, N, et al., 2004 | **DNA Microarray** | Iacobuzio-Donahue, CA, et al., 2003 |
|  |  |  |  |  |  |  |  | **DNA Microarray** | Lowe, AW, et al., 2007 |
|  |  |  |  |  |  |  |  | **IHC** | Karanjawala, ZE, et al., 2008 |
| **10.** | **F3** | **Coagulation factor III (thromboplastin, tissue factor)** | **Blood:** Drake, TA et al., 1989  **Hemodialysis fluid:** Molina, H, et al., 2005  **Tear:** de Souza, GA, et al., 2006  **Plasma membrane:** Carson, SD et al., 1985, Chen, VM et al., 2006 | **IHC** | Khorana, AA et al., 2007 | **NA** |  | **SAGE** | Hustinx, SR, et al., 2004 |
|  |  |  |  |  |  |  |  | **ELISA, other assays** | Haas, SL et al., 2006 |
| **11.** | **FSCN1** | **Fascin homolog 1, actin-bundling protein (Strongylocentrotus purpuratus)** | **Blood:** Sonderbye, L, et al., 1998  **Plasma membrane:** Lin, XH, et al., 1996, Tsai, WC, et al., 2007, Tsai, WC, et al., 2007 | **IHC** | Maitra, A, et al., 2003 | **RT-PCR, IHC** | Yamaguchi, H et al., 2007 | **ICAT** | Chen, R, et al., 2005 |
|  |  |  |  | **IHC** | Maitra, A et al., 2002 | **DNA Microarray** | Sato, N, et al., 2004 | **2D gel, IHC** | Lu, Z, et al., 2004 |
|  |  |  |  |  |  |  |  | **Western Blot** | Crnogorac-Jurcevic, T, et al., 2005 |
|  |  |  |  |  |  |  |  | **DNA Microarray** | Iacobuzio-Donahue, CA, et al., 2003 |
|  |  |  |  |  |  |  |  | **DNA Microarray** | Logsdon, CD, et al., 2003 |
|  |  |  |  |  |  |  |  | **DNA Microarray** | Nakamura, T, et al., 2004 |
|  |  |  |  |  |  |  |  | **DNA Microarray, IHC** | Iacobuzio-Donahue, CA, et al., 2002 |
| **12.** | **MMP7** | **Matrix metallopeptidase 7 (matrilysin, uterine)** | **Serum:** Laszlo, A, et al., 1990, Maurel, J, et al., 2007  **Seminal Plasma:**  Pilch, B, et al., 2006  **Plasma membrane:** Nishikawa, N, et al., 2006 | **IHC** | Li, YJ, et al., 2005 | **IHC** | Nishikawa, N, et al., 2006 | **DNA Microarray** | Iacobuzio-Donahue, CA, et al., 2003 |
|  |  |  |  | **DNA Microarray** | Buchholz, M, et al., 2005 |  |  | **IHC** | Nishikawa, N, et al., 2006 |
|  |  |  |  |  |  |  |  | **DNA Microarray** | Crnogorac-Jurcevic, T, et al., 2002 |
|  |  |  |  |  |  |  |  | **SAGE** | Hustinx, SR, et al., 2004 |
|  |  |  |  |  |  |  |  | **DNA Microarray** | Iacobuzio-Donahue, CA, et al., 2002 |
|  |  |  |  |  |  |  |  | **IHC** | Grutzmann, R, et al., 2004 |
|  |  |  |  |  |  |  |  | **IHC** | Li, YJ, et al., 2005 |
|  |  |  |  |  |  |  |  | **DNA Microarray** | Laurell, H, et al., 2006 |
|  |  |  |  |  |  |  |  | **Northern Blot, ISH** | Bramhall, SR, et al., 1997 |
| **13.** | **MST1R** | **Macrophage stimulating 1 receptor (c-met-related tyrosine kinase)** | **Plasma membrane:** Sakamoto, O et al., 1997, Gaudino, G et al., 1994, Wang, MH et al., 1994, Thomas, RM et al., 2007 | **Western Blot, IHC** | Thomas, RM, et al., 2007 | **NA** |  | **DNA Microarray** | Logsdon, CD, et al., 2003 |
|  |  |  |  | **IHC** | Camp, ER et al., 2007 |  |  | **DNA Microarray** | Johnson, SK, et al., 2006 |
|  |  |  |  |  |  |  |  | **DNA Microarray** | Lowe, AW, et al., 2007 |
|  |  |  |  |  |  |  |  | **IHC** | Camp, ER, et al., 2007 |
|  |  |  |  |  |  |  |  | **IHC** | Thomas, RM, et al., 2007 |
| **14.** | **MUC17** | **Mucin 17, cell surface associated** | **Plasma membrane:** Moniaux, N et al., 2006 | **ISH, IHC** | Park, HU, et al., 2003 | **NA** |  | **RT-PCR, IHC** | Moniaux, N, et al., 2006 |
|  |  |  |  |  |  |  |  | **ISH, IHC** | Park, HU, et al., 2003 |
| **15.** | **MUC4** | **Mucin 4, cell surface associated** | **Saliva:** Liu, B, et al., 1998  **Tear:** de Souza, GA, et al., 2006, Spurr-Michaud, S, et al., 2007  **Plasma membrane:** Ramsauer, VP, et al., 2003, Moniaux, N et al., 1999, Alameda, F, et al., 2007, Hu, YP et al., 2003, Duraisamy, S, et al., 2006, Zhang, J, et al., 2006, Park, HU, et al., 2003 | **ISH, IHC** | Park, HU, et al., 2003 | **IHC** | Kanno, A, et al., 2006 | **DNA Microarray** | Iacobuzio-Donahue, CA, et al., 2003 |
|  |  |  |  | **IHC** | Swartz, MJ, et al., 2002 |  |  | **RT-PCR** | Li, XH, et al., 2005 |
|  |  |  |  |  |  |  |  | **RT-PCR, IHC** | Andrianifahanana, M et al., 2006 |
|  |  |  |  |  |  |  |  | **DNA Microaray** | Segara, D, et al., 2005 |
|  |  |  |  |  |  |  |  | **ISH, IHC** | Park, HU, et al., 2003 |
|  |  |  |  |  |  |  |  | **RT-PCR** | Andrianifahanana, M, et al., 2001 |
|  |  |  |  |  |  |  |  | **IHC** | Saitou, M, et al., 2005 |
|  |  |  |  |  |  |  |  | **IHC** | Swartz, MJ, et al., 2002 |
|  |  |  |  |  |  |  |  | **IHC** | Kosmahl, M et al., 2004 |
| **16.** | **MUC6** | **Mucin 6, oligomeric mucus/gel-forming** | **Seminal Plasma:** Pilch, B, et al., 2006 | **DNA Microarray, RT-PCR, IHC** | Prasad, NB, et al., 2005 | **NA** |  | **IHC** | Bartman, AE et al., 1998 |
| **17.** | **S100P** | **S100 calcium binding protein P** | **Pancreatic Juice**: Gronborg, M, et al., 2004  **Tear:** de Souza, GA, et al., 2006  **Plasma membrane:** Koltzscher, M et al., 2003 | **RT-PCR** | Ohuchida, K et al., 2006 | **RT-PCR** | Ohuchida, K, et al., 2006 | **DNA Microaray, RT-PCR** | Fukushima, N, et al., 2004 |
|  |  |  |  | **DNA Microarray, RT-PCR** | Buchholz, M, et al., 2005 |  |  | **DNA Microarray** | Lowe, AW, et al., 2007 |
|  |  |  |  | **RT-PCR, IHC** | Dowen, SE et al., 2005 | **DNA Microarray, RT-PCR** | Sato, N, et al., 2004 | **DNA Microarray** | Iacobuzio-Donahue, CA, et al., 2002 |
|  |  |  |  | **DNA Microarray, RT-PCR** | Prasad, NB, et al., 2005 |  |  | **DNA Microaray, IHC** | Crnogorac-Jurcevic, T, et al., 2003 |
|  |  |  |  |  | | **DNA Microarray** | Terris, B, et al., 2002 | **DNA Microaray, RT-PCR** | Nakamura, T, et al., 2004 |
|  |  |  |  |  |  |  |  | **DNA Microarray** | Iacobuzio-Donahue, CA, et al., 2003 |
|  |  |  |  |  |  |  |  | **DNA Microarray** | Iacobuzio-Donahue, CA, et al., 2003 |
|  |  |  |  |  |  |  |  | **RT-PCR, IHC** | Logsdon, CD, et al., 2003 |
|  |  |  |  |  |  |  |  | **DNA Microarray** | Segara, D, et al., 2005 |
|  |  |  |  |  |  |  |  | **DNA Microarray** | Johnson, SK, et al., 2006 |
|  |  |  |  |  |  |  |  | **DNA Microarray** | Friess, H, et al., 2003 |
|  |  |  |  |  |  |  |  | **DNA Microarray, RT-PCR** | Buchholz, M, et al., 2005 |
|  |  |  |  |  |  |  |  | **RT-PCR** | Dowen, SE, et al., 2005 |
|  |  |  |  |  |  |  |  | **DNA Microarray** | Grutzmann, R, et al., 2004 |
|  |  |  |  |  |  |  |  | **DNA Microarray, RT-PCR, Northern Blot** | Jin, G, et al., 2005 |
|  |  |  |  |  |  |  |  | **DNA Microarray** | Pfeffer, F, et al., 2004 |
|  |  |  |  |  |  |  |  | **DNA Microarray** | Crnogorac-Jurcevic, T, et al., 2003 |
| **18.** | **SERPINB5** | **Serpin peptidase inhibitor, clade B (ovalbumin), member 5** | **Seminal Plasma:** Pilch, B, et al., 2006  **Tear:** de Souza, GA, et al., 2006  **Plasma membrane:** Sager, R, et al., 1997 | **IHC** | Maass, N, et al., 2001 | **DNA Microarray** | Sato, N, et al., 2004 | **DNA Microarray** | Logsdon, CD, et al., 2003 |
|  |  |  |  | **IHC** | Cao, D, et al., 2007 |  |  | **IHC** | Maass, N, et al., 2001 |
|  |  |  |  |  |  |  |  | **IHC** | Cao, D, et al., 2007 |
|  |  |  |  |  |  |  |  | **IHC** | Lim, YJ, et al., 2004 |
|  |  |  |  |  |  |  |  | **IHC** | Ohike, N, et al., 2003 |
|  |  |  |  |  |  |  |  | **DNA Microarray** | Pfeffer, F, et al., 2004 |
|  |  |  |  |  |  |  |  |  |  |
|  |  |  |  |  |  |  |  |  |  |
| **19.** | **SFN** | **Stratifin** | **Cerebrospinal fluid:** Burgess, JA et al., 2006  **Seminal plasma:** Pilch, B, et al., 2006  **Synovial fluid:** Kilani, RT et al., 2007  **Plasma:** States, DJ, et al., 2006 **Tear:** de Souza, GA, et al., 2006  **Plasma membrane:** Adachi, J, et al., 2006, Hustinx, SR et al., 2005 | **IHC** | Maitra, A, et al., 2003 | **DNA Microarray** | Sato, N, et al., 2004 | **IHC** | Hustinx, SR, et al., 2005 |
|  |  |  |  | **DNA Microarray** | Prasad, NB, et al., 2005 |  |  | **DNA Microarray** | Lowe, AW, et al., 2007 |
|  |  |  |  |  |  |  |  | **DNA Micraoarray** | Friess, H, et al., 2003 |
|  |  |  |  |  |  |  |  | **DNA Microarray, IHC** | Iacobuzio-Donahue, CA, et al., 2003 |
|  |  |  |  |  |  |  |  | **DNA Microarray, RT-PCR, IHC** | Logsdon, CD, et al., 2003 |
|  |  |  |  |  |  |  |  | **DNA Microarray, RT-PCR** | Johnson, SK, et al., 2006 |
|  |  |  |  |  |  |  |  | **DNA Microarray, RT-PCR, IHC, Western Blot** | Guweidhi, A et al., 2004 |
|  |  |  |  |  |  |  |  | **DNA Microarray** | Pfeffer, F, et al., 2004 |
|  |  |  |  |  |  |  |  | **DNA Microarray** | Iacobuzio-Donahue, CA, et al., 2003 |
|  |  |  |  |  |  |  |  | **DNA Microarray** | Nakamura, T, et al., 2004 |
| **20.** | **TFF1** | **Trefoil factor 1** | **Serum:** Vestergaard, EM, et al., 2004, Miyashita, S, et al., 1994, Gronbaek, H, et al., 2006  **Urine:** Adachi, J, et al., 2006,Chenard, MP, et al., 2004,Miyashita, S, et al., 1994 | **DNA Microarray** | Buchholz, M, et al., 2005 | **DNA Microarray, IHC** | Terris, B, et al., 2002 | **SAGE, IHC** | Terris, B, et al., 2002 |
|  |  |  |  | **DNA Microarray, RT-PCR, IHC** | Prasad, NB, et al., 2005 |  |  | **DNA Microarray** | Friess, H, et al., 2003 |
|  |  |  |  |  |  |  |  | **DNA Microarray** | Buchholz, M, et al., 2005 |
| **21.** | **TFF3** | **Trefoil factor 3 (intestinal)** | **Hemodialysis Fluid:** Molina, H, et al., 2005  **Plasma membrane:** Terris, B, et al., 2002 | **NA** |  | **DNA Microarray, IHC** | Terris, B, et al., 2002 |  | Lowe, AW, et al., 2007 |
|  |  |  |  |  |  |  |  | **SAGE, IHC** | Terris, B, et al., 2002 |
|  |  |  |  |  |  | **DNA Microarray** | Sato, N, et al., 2004 | **DNA Microarray** | Crnogorac-Jurcevic, T, et al., 2003 |
|  |  |  |  |  |  |  |  | **DNA Microarray** | Johnson, SK, et al., 2006 |
|  |  |  |  |  |  |  |  | **DNA Microarray** | Maitra, A, et al., 2003 |
|  |  |  |  |  |  |  |  |  |  |
| **22.** | **TIMP1** | **TIMP metallopeptidase inhibitor 1** | **Serum:** Zhou, W, et al., 1998  **Plasma:** Yukawa, N et al., 2007, States, DJ, et al., 2006, Sorensen, NM, et al., 2007, Caspersen, MB, et al., 2007  **Urine:** Adachi, J, et al., 2006 **Cerebrospinla fluid:** Zougman, A, et al., 2008  **Seminal plasma:** Pilch, B, et al., 2006, Baumgart, E et al., 2002  **Gingival crevicular fluid:** Emingil, G, et al., 2006  **Saliva:** Ramachandran, P, et al., 2006  **Tear:** de Souza, GA, et al., 2006 | **NA** |  | **DNA Microarray, IHC** | Sato, N, et al., 2004 | **DNA Microarray** | Iacobuzio-Donahue, CA, et al., 2003 |
|  |  |  |  |  |  |  |  | **DNA Microarray** | Iacobuzio-Donahue, CA, et al., 2003 |
|  |  |  |  |  |  |  |  | **DNA Microarray** | Jin, G, et al., 2005 |
|  |  |  |  |  |  |  |  | **DNA Microarray** | Laurell, H, et al., 2006 |
|  |  |  |  |  |  |  |  | **Northern Blot, ISH** | Bramhall, SR, et al., 1997 |
|  |  |  |  |  |  |  |  | **ELISA, Northern Blot** | Zhou, W, et al., 1998 |
|  |  |  |  |  |  |  |  | **DNA Microarray** | Crnogorac-Jurcevic, T et al., 2001 |
| **23.** | **PLAU** | **Plasminogen activator, urokinase** | **Serum:** Miyake, H et al., 1999, States, DJ, et al., 2006  **Plasma membrane:** Harvey, SR, et al., 2003, Ueshima, S et al., 1999, Nielsen, A et al., 2005, Mukhina, S, et al., 2000 | **IHC** | Harvey, SR, et al., 2003 | **NA** |  | **DNA Microarray** | Lowe, AW, et al., 2007 |
|  |  |  |  |  |  |  |  | **DNA Microarray** | Crnogorac-Jurcevic, T, et al., 2003 |
|  |  |  |  |  |  |  |  | **DNA Microarray** | Iacobuzio-Donahue, CA, et al., 2003 |
|  |  |  |  |  |  |  |  | **RT-PCR, IHC** | Nielsen, A, et al., 2005 |
|  |  |  |  |  |  |  |  | **DNA Microarray** | Laurell, H, et al., 2006 |
|  |  |  |  |  |  |  |  | **DNA Microarray** | Iacobuzio-Donahue, CA, et al., 2002 |
|  |  |  |  |  |  |  |  | **Western Blot, IHC** | Wang, W et al., 1999 |
|  |  |  |  |  |  |  |  | **ISH, IHC** | Harvey, SR, et al., 2003 |
|  |  |  |  |  |  |  |  | **DNA Microarray** | Nakamura, T, et al., 2004 |
|  |  |  |  |  |  |  |  | **IHC** | Paciucci, R, et al., 1998 |

**Table E**

**Partial list of molecules overexpressed in chronic pancreatitis along with their expression status in pancreatic ductal adenocarcinoma**

|  | **Gene Symbol** | **Protein Name** | **Whether detected in body fluids/plasma membrane** | Chronic pancreatitis | | | PDAC | | |
| --- | --- | --- | --- | --- | --- | --- | --- | --- | --- |
|  |  |  |  | **Method** | **Cell type** | **Reference** | **Method** | **Cell type** | **Reference** |
| **1.** | **EPHA3** | **EPH receptor A3** | **Plasma membrane:** Boyd, AW et al., 1992 | **DNA Microarray** | **Stroma** | Fukushima, N, et al., 2005 | **DNA Microarray** | **Stroma** | Fukushima, N, et al., 2005 |
| **2.** | **FBN1** | **Fibrillin 1** | **Cerebrospinal fluid:** Zougman, A, et al., 2008 | **DNA Microarray** | **Stroma** | Binkley, CE et al., 2004 | **DNA Microarray** | **Stroma** | Binkley, CE, et al., 2004 |
|  |  |  |  | **ICAT** |  | Yesudian, PD et al., 2007 | **DNA Microarray** |  | Lowe, AW, et al., 2007 |
|  |  |  |  |  |  |  | **DNA Microarray** |  | Johnson, SK, et al., 2006 |
|  |  |  |  |  |  |  | **ICAT** |  | Chen, R, et al., 2005 |
| **3.** | **IL8** | **Interleukin 8** | **Serum:** Bellone, G et al., 2006, Goetzl, L et al., 2002, Morelli, SS et al., 2008, Yang, SQ et al., 2008, Yasui, T et al., 2008  **Plasma:** Chu, CJ et al., 2007 | **IHC** | **Nerves** | Di Sebastiano, P et al., 2000 | **IHC, ELISA** | **NA** | Bellone, G, et al., 2006 |
|  |  |  |  | **ISH** | **Stroma** | Saurer, L et al., 2000 | **DNA Microarray** | **NA** | Iacobuzio-Donahue, CA, et al., 2003 |
|  |  |  |  | **DNA Microarry** |  | Rogers, CD, et al., 2006 | **DNA Microarry** |  | Rogers, CD, et al., 2006 |
|  |  |  |  | **DNA Microarray** |  | Farrow, B et al., 2004 | **DNA Microarray** |  | Iacobuzio-Donahue, CA, et al., 2002 |
|  |  |  |  | **Other assays** |  | Zeh, HJ et al., 2005 | **DNA Miroarray** |  | Segara, D, et al., 2005 |
|  |  |  |  |  |  |  | **RT-PCR** |  | Bellone, G, et al., 2006 |
|  |  |  |  |  |  |  | **Other assays** |  | Zeh, HJ, et al., 2005 |
| **4.** | **FGA** | **Fibrinogen alpha chain** | **Hemodialysis fluid:** Molina, H, et al., 2005  **Serum:** States, DJ, et al., 2006  **Pancreatic fluid:** Gronborg, M, et al., 2004  **Tear:** de Souza, GA, et al., 2006 **Cerebrospinal fluid:** Zougman, A, et al., 2008 | **DNA Microarray** | **NA** | Rogers, CD, et al., 2006 | **DNA Microarray** | **Stroma** | Fukushima, N, et al., 2005 |
|  |  |  |  |  |  |  | **DNA Microarray** | **NA** | Rogers, CD, et al., 2006 |
| **5.** | **FGG** | **Fibrinogen gamma chain** | **Serum:** Bloomston, M et al., 2006, States, DJ, et al., 2006  **Pancreatic fluid:** Gronborg, M, et al., 2004, Chen, R, et al., 2006  **Bile:** Kristiansen, TZ, et al., 2004  **Cerebrospinal fluid:** Zougman, A, et al., 2008, Burgess, JA, et al., 2006  **Tear:** de Souza, GA, et al., 2006  **Saliva:** Ramachandran, P, et al., 2006 | **ICAT** | **NA** | Chen, R, et al., 2007 | **IHC, 2D Gel, Other assays** | **Stroma** | Bloomston, M, et al., 2006 |
|  |  |  |  |  |  |  | **ICAT** |  | Chen, R, et al., 2007 |
|  |  |  |  |  |  |  | **ICAT** |  | Chen, R, et al., 2005 |
|  |  |  |  |  |  |  | **ICAT** |  | Chen, R, et al., 2006 |
| **6.** | **A1BG** | Alpha-1-B glycoprotein | Plasma: Gahne, B et al., 1987, States, DJ, et al., 2006  Serum: Adkins, JN et al., 2002  Cerebrospinal fluid: Burgess, JA, et al., 2006  **Saliva:** Ramachandran, P, et al., 2006 | **ICAT** |  | Chen, R, et al., 2007 | **ICAT**  **Not Changed** |  | Chen, R, et al., 2007 |
| **7.** | **CCL3** | MIP-1 alpha | **Serum:** Zeh, HJ, et al., 2005, Adkins, JN, et al., 2002  **Cerebrospinal fluid:** Letendre, SL et al., 1999 | **Other assays**  **(Cytokine Multiplex Assay, LabMap)** |  | Zeh, HJ, et al., 2005 | **Other assays (Cytokine Multiplex Assay, LabMap)**  **Not changed** |  | Zeh, HJ, et al., 2005 |
|  |  |  |  | **RT-PCR** |  | Singh, L et al., 2007 |  |  |  |
|  |  |  |  | **Northern Blot** |  | Goecke, H et al., 2000 |  |  |  |
| **8.** | **CCL4** | MIP-1 beta | Serum: Zeh, HJ, et al., 2005  Plasma: Anderson, NL, et al., 2004  **Cerebrospinal fluid:** Letendre, SL, et al., 1999 | **Other assays**  **(Cytokine Multiplex Assay, LabMap)** |  | Zeh, HJ, et al., 2005 | **Other assays**  **(Cytokine Multiplex Assay, LabMap)**  **Not changed** |  | Zeh, HJ, et al., 2005 |
|  |  |  |  | **RT-PCR** |  | Singh, L, et al., 2007 |  |  |  |
| **9.** | **SOD3** | Superoxide dismutase 3 | **Cerebrospinal fluid:** Burgess, JA, et al., 2006  **Serum:** Folz, RJ et al., 1994  **Plasma:** Saitoh, D et al., 2001 Adachi, T et al., 1998 | **ICAT** |  | Chen, R, et al., 2007 | **DNA Microarray**  **Down regulated** |  | Logsdon, CD, et al., 2003 |
| **10.** | **TAC1** | Tachykinin1 | **Plasma:** Shang, YX et al., 2003  **Serum:** Bruno, G et al., 2003  **Nasal lavage fluid:** Cho, YS et al., 2003 | **IHC** | **Nerves** | Di Sebastiano, P, et al., 2000 |  |  | NA |

**Table F**

**Partial list of molecules overexpressed in the stroma associated with pancreatic cancer**

|  | **Gene Symbol** | **Protein name** | **Whether detected in body fluids/plasma membrane** | Pancreatic cancer | | | |
| --- | --- | --- | --- | --- | --- | --- | --- |
|  |  |  |  | Method | Cell type | **Cancer tissue** | **Reference** |
| **1.** | **LGALS1** | **Galectin 1** | **Plasma:** Anderson, NL, et al., 2004  **Hemodialysis fluid:** Molina, H, et al., 2005  **Cerebrospinal fluid:** Zougman, A, et al., 2008  **Plasma membrane:** Akimoto, Y et al., 1995 | **IHC** | **Stroma** | Not upregulated in tumor cells | Shen, J et al., 2004 |
|  |  |  |  | **ISH, IHC** | **Nerves** | Not upregulated in tumor cells | Berberat, PO, et al., 2001 |
| **2.** | **CTSB** | **Cathepsin B** | **Plasma:** Anderson, NL, et al., 2004  **Seminal plasma:** Pilch, B, et al., 2006  **Cerebrospinal fluid:** Zougman, A, et al., 2008  **Tear**: de Souza, GA, et al., 2006 | **IHC** | **Fibroblast** | Invasive ductal adenocarcinoma | Niedergethmann, M et al., 2004 |
| **3.** | **FASLG** | **Fas ligand (TNF superfamily, member 6)** | **Plasma:** States, DJ, et al., 2006  **Plasma membrane:** Ungefroren, H et al., 1998, Suda, T et al., 1993, Albanese, J et al., 2000 | **IHC** | **Lymphocytes** | Invasive ductal adenocarcinoma | Satoh, K et al., 1999 |
|  |  |  |  | **IHC** | **Stroma** | Invasive ductal adenocarcinoma | Boltze, C et al., 2002 |
| **4.** | **SERPINH1** | **Serpin peptidase inhibitor, clade H (heat shock protein 47), member 1, (collagen binding protein 1)** | **Plasma membrane:** Hebert, C et al., 1999  **Serum:** Yokota, S et al., 2003 | **IHC** | Fibroblasts | PanIN-3 | Maitra, A, et al., 2002 |
| **5.** | **MMP2** | **Matrix metallopeptidase 2 (gelatinase A, 72kDa gelatinase, 72kDa type IV collagenase)** | **Plasma:** States, DJ, et al., 2006  **Seminal plasma:** Shimokawa Ki, K et al., 2002, Pilch, B, et al., 2006  **Cerebrospinal fluid:** Zougman, A, et al., 2008  **Plasma membrane:** Bramhall, SR, et al., 1997 | **IHC** | **Stroma** | Invasive ductal adenocarcinoma | Gurevich, LE, 2003 |
|  |  |  |  | **IHC** | **Fibroblasts** | Invasive ductal adenocarcinoma | Schneiderhan, W, et al., 2007 |
|  |  |  |  | IHC, ISH | Stroma | Invasive ductal adenocarcinoma | Bramhall, SR, et al., 1997 |
|  |  |  |  | **DNA Mciroarray** | Stroma | Invasive ductal adenocarcinoma | Binkley, CE, et al., 2004 |
|  |  |  |  | **ISH** | Stroma | Invasive ductal adenocarcinoma | Iacobuzio-Donahue, CA et al., 2002 |
|  |  |  |  | **IHC** | Stroma | Invasive ductal adenocarcinoma | Ellenrieder, V et al., 2000 |
| **6.** | **POSTN** | **Periostin, osteoblast specific factor** | **Serum:** Sasaki, H, et al., 2003, Sasaki, H, et al., 2001, Sasaki, H, et al., 2002, Baril, P, et al., 2007 | **IHC** | Stroma | Invasive ductal adenocarcinoma | Baril, P, et al., 2007 |
|  |  |  |  | **DNA Mciroarray** | Stroma | Invasive ductal adenocarcinoma | Binkley, CE, et al., 2004 |
|  |  |  |  | **IHC** | Stroma | Invasive ductal adenocarcinoma | Erkan, M, et al., 2007 |
| **7.** | **THBS1** | **Thrombospondin 1** | **Plasma:** States, DJ, et al., 2006  **Serum:** Sheng, S, et al., 2006  **Saliva:** Ramachandran, P, et al., 2006  **Cerebrospinal fluid:** Burgess, JA, et al., 2006  **Seminal plasma:** Pilch, B, et al., 2006 | **IHC** | Stroma | Invasive ductal adenocarcinoma | Qian, X, et al., 2001 |
|  |  |  |  | **ISH** | Stroma | Invasive ductal adenocarcinoma | Iacobuzio-Donahue, CA, et al., 2002 |
| **8.** | **TNC** | **Tenascin C (hexabrachion)** | Serum: Degen, M et al., 2008, Schenk, S et al., 1995, Pauli, C et al., 2002  Plasma: Takeda, A et al., 2007  **Cerebrospinal fluid:** Suzuki, H et al., 2008 | IHC | Stroma | Invasive ductal adenocarcinoma; PanIN-1A and PanIN-1B; PanIN-2; PanIN-3 | Esposito, I, et al., 2006 |
|  |  |  |  | **IHC** | Stroma | Invasive ductal adenocarcinoma | Juuti, A et al., 2004 |
| **9.** | **VCAN** | Versican | **Cerebrospinal fluid:** Zougman, A, et al., 2008  **Hemodialysis fluid:** Molina, H, et al., 2005 | **IHC** | Fibroblasts | Invasive ductal adenocarcinoma | Koninger, J et al., 2004 |
| **10.** | **GPC1** | **Glypican 1** | **Cerebrospinal fluid:** Zougman, A, et al., 2008  **Seminal plasma:** Pilch, B, et al., 2006  **Plasma membrane:** David, G et al., 1990, Schofield, KP et al., 1999, Lories, V et al., 1992 | **IHC, ISH** | Fibroblasts | Invasive ductal adenocarcinoma | Kleeff, J et al., 1998 |
| **11.** | **FGF5** | **Fibroblast growth factor 5** |  | **IHC** | Muscle, | Invasive ductal adenocarcinoma | Kornmann, M et al., 1997 |
|  |  |  |  | **ISH** | Macrophages | Invasive ductal adenocarcinoma | Kornmann, M, et al., 1997 |
| **12.** | **MMP11** | **Matrix metallopeptidase 11 (stromelysin 3)** | **Serum:** Tirumalai, RS, et al., 2003, Yang, YH, et al., 2008 | **IHC** | Stroma | Invasive ductal adenocarcinoma | von Marschall, Z, et al., 1998 |
|  |  |  |  | **ISH** | Stroma | Invasive ductal adenocarcinoma | Iacobuzio-Donahue, CA, et al., 2002 |
| **13.** | **DCN** | **Decorin** | **Cerebrospinal fluid:** Zougman, A, et al., 2008 | **IHC** | Fibroblast | Invasive ductal adenocarcinoma | Koninger, J, et al., 2004 |
| **14.** | **SPARC** | **Secreted protein, acidic, cysteine-rich (osteonectin)** | **Cerebrospinal fluid:** Zougman, A, et al., 2008  **Plasma:** States, DJ, et al., 2006, Takahashi, M et al., 2001  **Serum:** Guweidhi, A et al., 2005 | **IHC** | Fibroblasts | Invasive ductal adenocarcinoma | Guweidhi, A, et al., 2005 |
|  |  |  |  | **DNA Microarray** | Stroma | Invasive ductal adenocarcinoma | Binkley, CE, et al., 2004 |
| **15.** | **SPP1** | **Secreted phosphoprotein 1 (osteopontin, bone sialoprotein I, early T-lymphocyte activation 1)** | **Serum:** Koopmann, J, et al., 2006, Kang, SY, et al., 2007, Koopmann, J, et al., 2004, Kolb, A, et al., 2005  **Urine:** Adachi, J, et al., 2006, Christensen, B, et al., 2008  **Cerebrospinal fluid:** Zougman, A, et al., 2008, 16199891),  **Hemodialysis fluid:** Molina, H, et al., 2005 | **IHC** | Macrophages | Invasive ductal adenocarcinoma | Koopmann, J, et al., 2004 |
|  |  |  |  | **DNA Microarray** | Stroma | Invasive ductal adenocarcinoma | Fukushima, N, et al., 2005 |
| **16.** | **CTSL1** | **Cathepsin L1** | **Serum:** Lang, TH et al., 2004, Dong, M et al., 2007 | **IHC** | Macrophages | Invasive ductal adenocarcinoma | Niedergethmann, M, et al., 2004 |

**Table G**

**Partial list of molecules showing elevated expression in different subtypes of pancreatic cancer**

|  | **Gene symbol** | **Protein name** | **Whether detected in body fluids/plasma membrane** | **Invasive ductal adenocarcinoma** | | **Endocrine neoplasms** | | **Mucinous cystic neoplasms** | |
| --- | --- | --- | --- | --- | --- | --- | --- | --- | --- |
|  |  |  |  | **Method** | **Reference** | **Method** | **Reference** | **Method** | **Reference** |
| **1.** | **AXL** | **AXL receptor tyrosine kinase** | **Cerebrospinal fluid:** Zougman, A, et al., 2008  **Plasma membrane:** O'Bryan, JP et al., 1995, Yanagita, M et al., 2001 | **DNA microarray** | Laurell, H, et al., 2006 | DNA microarray | Lowe, AW, et al., 2007 | **DNA microarray** | Fukushima, N, et al., 2004 |
| **2.** | **THBS2** | **Thrombospondin 2** | **Seminal Plasma:** Pilch, B, et al., 2006  **Cerebrospinal Fluid:** Zougman, A, et al., 2008  **Plasma:** States, DJ, et al., 2006 | **Western Blot** | Crnogorac-Jurcevic, T, et al., 2005 |  | NA | **DNA Microarray** | Fukushima, N, et al., 2004 |
|  |  |  |  | **DNA Microarray** | Lowe, AW, et al., 2007 |  |  |  |  |
|  |  |  |  | **DNA Microarray** | Nakamura, T, et al., 2004 |  |  |  |  |
|  |  |  |  | **DNA Microarray** | Iacobuzio-Donahue, CA, et al., 2003 |  |  |  |  |
|  |  |  |  | **DNA Microarray** | Friess, H, et al., 2003 |  |  |  |  |
|  |  |  |  | **DNA Microarray** | Iacobuzio-Donahue, CA, et al., 2002 |  |  |  |  |
|  |  |  |  | **DNA Microarray, RT-PCR, IHC** | Binkley, CE, et al., 2004 |  |  |  |  |
| **3.** | **CD74** | **CD74 molecule, major histocompatibility complex, class II invariant chain** | **Plasma membrane:** Mawby, WJ et al., 1994, Claesson, L et al., 1983, Roche, PA et al., 1993, Claesson, L et al., 1983 | **IHC, SAGE** | Hustinx, SR, et al., 2004 |  | NA |  | NA |
|  |  |  |  | **DNA Microarray** | Laurell, H, et al., 2006 |  |  |  |  |
|  |  |  |  | **IHC** | Koide, N et al., 2006 |  |  |  |  |
|  |  |  |  | **DNA Microarray** | Binkley, CE, et al., 2004 |  |  |  |  |
| **4.** | **SCG5** | **Secretogranin V (7B2 protein)** | **Cerebrospinal Fluid:** Zougman, A, et al., 2008 |  | NA | **Northern Blot, IHC** | Gherzi, R et al., 1994 |  | NA |
| **5.** | **PTPRN2** | **Protein tyrosine phosphatase, receptor type, N polypeptide 2** | **Plasma membrane:** Cui, L et al., 1996, Lan, MS et al., 1994 |  | NA | **DNA Microarray** | Lowe, AW, et al., 2007 |  | NA |
| **6.** | **BIN1** | **Bridging integrator 1** | **Plasma**: States, DJ, et al., 2006  **Plasma membrane:** Ramjaun, AR et al., 1999 |  | NA | **DNA Microarray, RT-PCR, IHC** | Capurso, G et al., 2006 |  | NA |
| **7.** | **IGFBP1** | **Insulin-like growth factor binding protein 1** | Serum: Karna, E et al., 2002, Rajaram, S et al., 1997, Stone, S et al., 2003, Akturk, M et al., 2007  Milk: Suikkari, AM, 1989  **Plasma:** Anderson, NL, et al., 2004  **Amniotic fluid**: Bell, SC et al., 1989  **Urine**: Lee, DY et al., 1994 | **Western Blot, Other assays** | Karna, E, et al., 2002 | **DNA Microarray** | Hansel, DE et al., 2004 |  | NA |
|  |  |  |  | **RT-PCR** | Gress, TM, et al., 1997 |  |  |  |  |
| **8.** | **LYZ** | **lysozyme (renal amyloidosis)** | **Seminal plasma:** Pilch, B, et al., 2006  **Pancreatic Fluid:** Gronborg, M, et al., 2004, Chen, R, et al., 2007  **Cerebrospinal fluid:** Zougman, A, et al., 2008  **Bile:** Kristiansen, TZ, et al., 2004  **Tear**: de Souza, GA, et al., 2006 | **SAGE** | Hustinx, SR, et al., 2004 | **DNA Microarray** | Hansel, DE, et al., 2004 |  | NA |
|  |  |  |  | **IHC** | Kim, JH et al., 1990 |  |  |  |  |
| **9.** | **MSH2** | **mutS homolog 2, colon cancer, nonpolyposis type 1** | **Plasma:** States, DJ, et al., 2006 |  | NA |  | NA | **IHC** | Luttges, J, et al., 2002 |
| **10.** | **STAR** | **Steroidogenic acute regulatory protein** |  |  | NA |  | NA | **DNA Microarray, RT-PCR** | Fukushima, N, et al., 2004 |

**References**

1. Gronborg M, Bunkenborg J, Kristiansen TZ, Jensen ON, Yeo CJ, et al. (2004) Comprehensive proteomic analysis of human pancreatic juice. J Proteome Res 3: 1042-1055.

2. Muller S, Goletz S, Packer N, Gooley A, Lawson AM, et al. (1997) Localization of O-glycosylation sites on glycopeptide fragments from lactation-associated MUC1. All putative sites within the tandem repeat are glycosylation targets in vivo. J Biol Chem 272: 24780-24793.

3. Storr SJ, Royle L, Chapman CJ, Hamid UM, Robertson JF, et al. (2008) The O-linked glycosylation of secretory/shed MUC1 from advanced breast cancer patient serum. Glycobiology.

4. Moreno M, Bontkes HJ, Scheper RJ, Kenemans P, Verheijen RH, et al. (2007) High level of MUC1 in serum of ovarian and breast cancer patients inhibits huHMFG-1 dependent cell-mediated cytotoxicity (ADCC). Cancer Lett 257: 47-55.

5. Gold DV, Modrak DE, Ying Z, Cardillo TM, Sharkey RM, et al. (2006) New MUC1 serum immunoassay differentiates pancreatic cancer from pancreatitis. J Clin Oncol 24: 252-258.

6. Wykes M, MacDonald KP, Tran M, Quin RJ, Xing PX, et al. (2002) MUC1 epithelial mucin (CD227) is expressed by activated dendritic cells. J Leukoc Biol 72: 692-701.

7. Tsutsumida H, Swanson BJ, Singh PK, Caffrey TC, Kitajima S, et al. (2006) RNA interference suppression of MUC1 reduces the growth rate and metastatic phenotype of human pancreatic cancer cells. Clin Cancer Res 12: 2976-2987.

8. Raina D, Ahmad R, Kumar S, Ren J, Yoshida K, et al. (2006) MUC1 oncoprotein blocks nuclear targeting of c-Abl in the apoptotic response to DNA damage. Embo J 25: 3774-3783.

9. Li Y, Ren J, Yu W, Li Q, Kuwahara H, et al. (2001) The epidermal growth factor receptor regulates interaction of the human DF3/MUC1 carcinoma antigen with c-Src and beta-catenin. J Biol Chem 276: 35239-35242.

10. Tajiri T, Tate G, Kunimura T, Inoue K, Mitsuya T, et al. (2004) Histologic and immunohistochemical comparison of intraductal tubular carcinoma, intraductal papillary-mucinous carcinoma, and ductal adenocarcinoma of the pancreas. Pancreas 29: 116-122.

11. Qu CF, Li Y, Song YJ, Rizvi SM, Raja C, et al. (2004) MUC1 expression in primary and metastatic pancreatic cancer cells for in vitro treatment by (213)Bi-C595 radioimmunoconjugate. Br J Cancer 91: 2086-2093.

12. Chhieng DC, Benson E, Eltoum I, Eloubeidi MA, Jhala N, et al. (2003) MUC1 and MUC2 expression in pancreatic ductal carcinoma obtained by fine-needle aspiration. Cancer 99: 365-371.

13. Luttges J, Zamboni G, Longnecker D, Kloppel G (2001) The immunohistochemical mucin expression pattern distinguishes different types of intraductal papillary mucinous neoplasms of the pancreas and determines their relationship to mucinous noncystic carcinoma and ductal adenocarcinoma. Am J Surg Pathol 25: 942-948.

14. Ohuchida K, Mizumoto K, Yamada D, Fujii K, Ishikawa N, et al. (2006) Quantitative analysis of MUC1 and MUC5AC mRNA in pancreatic juice for preoperative diagnosis of pancreatic cancer. Int J Cancer 118: 405-411.

15. Saitou M, Goto M, Horinouchi M, Tamada S, Nagata K, et al. (2005) MUC4 expression is a novel prognostic factor in patients with invasive ductal carcinoma of the pancreas. J Clin Pathol 58: 845-852.

16. Andrianifahanana M, Moniaux N, Schmied BM, Ringel J, Friess H, et al. (2001) Mucin (MUC) gene expression in human pancreatic adenocarcinoma and chronic pancreatitis: a potential role of MUC4 as a tumor marker of diagnostic significance. Clin Cancer Res 7: 4033-4040.

17. Hustinx SR, Cao D, Maitra A, Sato N, Martin ST, et al. (2004) Differentially expressed genes in pancreatic ductal adenocarcinomas identified through serial analysis of gene expression. Cancer Biol Ther 3: 1254-1261.

18. Pilch B, Mann M (2006) Large-scale and high-confidence proteomic analysis of human seminal plasma. Genome Biol 7: R40.

19. Maurel J, Nadal C, Garcia-Albeniz X, Gallego R, Carcereny E, et al. (2007) Serum matrix metalloproteinase 7 levels identifies poor prognosis advanced colorectal cancer patients. Int J Cancer 121: 1066-1071.

20. Laszlo A, Sohar I, Karacsonyi S, Petri A, Trojan I (1990) Activities of serum cathepsin (B, H and L) and metalloproteinase (MMP7-ase) in patients with gastrointestinal and bronchial malignant tumours. Acta Med Hung 47: 107-109.

21. Nishikawa N, Kimura Y, Okita K, Zembutsu H, Furuhata T, et al. (2006) Intraductal papillary mucinous neoplasms of the pancreas: an analysis of protein expression and clinical features. J Hepatobiliary Pancreat Surg 13: 327-335.

22. Iacobuzio-Donahue CA, Ashfaq R, Maitra A, Adsay NV, Shen-Ong GL, et al. (2003) Highly expressed genes in pancreatic ductal adenocarcinomas: a comprehensive characterization and comparison of the transcription profiles obtained from three major technologies. Cancer Res 63: 8614-8622.

23. Laurell H, Bouisson M, Berthelemy P, Rochaix P, Dejean S, et al. (2006) Identification of biomarkers of human pancreatic adenocarcinomas by expression profiling and validation with gene expression analysis in endoscopic ultrasound-guided fine needle aspiration samples. World J Gastroenterol 12: 3344-3351.

24. Crnogorac-Jurcevic T, Efthimiou E, Nielsen T, Loader J, Terris B, et al. (2002) Expression profiling of microdissected pancreatic adenocarcinomas. Oncogene 21: 4587-4594.

25. Iacobuzio-Donahue CA, Maitra A, Shen-Ong GL, van Heek T, Ashfaq R, et al. (2002) Discovery of novel tumor markers of pancreatic cancer using global gene expression technology. Am J Pathol 160: 1239-1249.

26. Bramhall SR, Neoptolemos JP, Stamp GW, Lemoine NR (1997) Imbalance of expression of matrix metalloproteinases (MMPs) and tissue inhibitors of the matrix metalloproteinases (TIMPs) in human pancreatic carcinoma. J Pathol 182: 347-355.

27. Li YJ, Wei ZM, Meng YX, Ji XR (2005) Beta-catenin up-regulates the expression of cyclinD1, c-myc and MMP-7 in human pancreatic cancer: relationships with carcinogenesis and metastasis. World J Gastroenterol 11: 2117-2123.

28. Sheng S, Chen D, Van Eyk JE (2006) Multidimensional liquid chromatography separation of intact proteins by chromatographic focusing and reversed phase of the human serum proteome: optimization and protein database. Mol Cell Proteomics 5: 26-34.

29. de Souza GA, Godoy LM, Mann M (2006) Identification of 491 proteins in the tear fluid proteome reveals a large number of proteases and protease inhibitors. Genome Biol 7: R72.

30. Neyraud E, Sayd T, Morzel M, Dransfield E (2006) Proteomic analysis of human whole and parotid salivas following stimulation by different tastes. J Proteome Res 5: 2474-2480.

31. Wang W, Kirsch T (2002) Retinoic acid stimulates annexin-mediated growth plate chondrocyte mineralization. J Cell Biol 157: 1061-1069.

32. Barwise JL, Walker JH (1996) Annexins II, IV, V and VI relocate in response to rises in intracellular calcium in human foreskin fibroblasts. J Cell Sci 109 (Pt 1): 247-255.

33. Tian R, Wei LM, Qin RY, Li Y, Du ZY, et al. (2008) Proteome analysis of human pancreatic ductal adenocarcinoma tissue using two-dimensional gel electrophoresis and tandem mass spectrometry for identification of disease-related proteins. Dig Dis Sci 53: 65-72.

34. Paciucci R, Tora M, Diaz VM, Real FX (1998) The plasminogen activator system in pancreas cancer: role of t-PA in the invasive potential in vitro. Oncogene 16: 625-633.

35. Esposito I, Penzel R, Chaib-Harrireche M, Barcena U, Bergmann F, et al. (2006) Tenascin C and annexin II expression in the process of pancreatic carcinogenesis. J Pathol 208: 673-685.

36. Chen R, Brentnall TA, Pan S, Cooke K, Moyes KW, et al. (2007) Quantitative proteomics analysis reveals that proteins differentially expressed in chronic pancreatitis are also frequently involved in pancreatic cancer. Mol Cell Proteomics 6: 1331-1342.

37. Chen R, Yi EC, Donohoe S, Pan S, Eng J, et al. (2005) Pancreatic cancer proteome: the proteins that underlie invasion, metastasis, and immunologic escape. Gastroenterology 129: 1187-1197.

38. Crnogorac-Jurcevic T, Gangeswaran R, Bhakta V, Capurso G, Lattimore S, et al. (2005) Proteomic analysis of chronic pancreatitis and pancreatic adenocarcinoma. Gastroenterology 129: 1454-1463.

39. Ortiz-Zapater E, Peiro S, Roda O, Corominas JM, Aguilar S, et al. (2007) Tissue plasminogen activator induces pancreatic cancer cell proliferation by a non-catalytic mechanism that requires extracellular signal-regulated kinase 1/2 activation through epidermal growth factor receptor and annexin A2. Am J Pathol 170: 1573-1584.

40. Nakamura T, Furukawa Y, Nakagawa H, Tsunoda T, Ohigashi H, et al. (2004) Genome-wide cDNA microarray analysis of gene expression profiles in pancreatic cancers using populations of tumor cells and normal ductal epithelial cells selected for purity by laser microdissection. Oncogene 23: 2385-2400.

41. Lopes RB, Gangeswaran R, McNeish IA, Wang Y, Lemoine NR (2007) Expression of the IAP protein family is dysregulated in pancreatic cancer cells and is important for resistance to chemotherapy. Int J Cancer 120: 2344-2352.

42. Satoh K, Kaneko K, Hirota M, Masamune A, Satoh A, et al. (2001) Expression of survivin is correlated with cancer cell apoptosis and is involved in the development of human pancreatic duct cell tumors. Cancer 92: 271-278.

43. Wei H, Wang C, Chen L (2006) Proliferating cell nuclear antigen, survivin, and CD34 expressions in pancreatic cancer and their correlation with hypoxia-inducible factor 1alpha. Pancreas 32: 159-163.

44. Qiao JG, Zhang YQ, Yin YC, Tan Z (2004) Expression of Survivin in pancreatic cancer and its correlation to expression of Bcl-2. World J Gastroenterol 10: 2759-2761.

45. Bhanot U, Heydrich R, Moller P, Hasel C (2006) Survivin expression in pancreatic intraepithelial neoplasia (PanIN): steady increase along the developmental stages of pancreatic ductal adenocarcinoma. Am J Surg Pathol 30: 754-759.

46. Sun HC, Qiu ZJ, Liu J, Sun J, Jiang T, et al. (2007) Expression of hypoxia-inducible factor-1 alpha and associated proteins in pancreatic ductal adenocarcinoma and their impact on prognosis. Int J Oncol 30: 1359-1367.

47. Satoh K, Kaneko K, Hirota M, Toyota T, Shimosegawa T (2000) The pattern of CPP32/caspase-3 expression reflects the biological behavior of the human pancreatic duct cell tumors. Pancreas 21: 352-357.

48. Carbone A, Rodeck U, Mauri FA, Sozzi M, Gaspari F, et al. (2005) Human pancreatic carcinoma cells secrete bioactive interleukin-18 after treatment with 5-fluorouracil: implications for anti-tumor immune response. Cancer Biol Ther 4: 231-241.

49. Virkajarvi N, Paakko P, Soini Y (1998) Apoptotic index and apoptosis influencing proteins bcl-2, mcl-1, bax and caspases 3, 6 and 8 in pancreatic carcinoma. Histopathology 33: 432-439.

50. Meggiato T, Calabrese F, De Cesare CM, Baliello E, Valente M, et al. (2003) C-JUN and CPP32 (CASPASE 3) in human pancreatic cancer: relation to cell proliferation and death. Pancreas 26: 65-70.

51. Grutzmann R, Pilarsky C, Ammerpohl O, Luttges J, Bohme A, et al. (2004) Gene expression profiling of microdissected pancreatic ductal carcinomas using high-density DNA microarrays. Neoplasia 6: 611-622.

52. Grutzmann R, Boriss H, Ammerpohl O, Luttges J, Kalthoff H, et al. (2005) Meta-analysis of microarray data on pancreatic cancer defines a set of commonly dysregulated genes. Oncogene 24: 5079-5088.

53. States DJ, Omenn GS, Blackwell TW, Fermin D, Eng J, et al. (2006) Challenges in deriving high-confidence protein identifications from data gathered by a HUPO plasma proteome collaborative study. Nat Biotechnol 24: 333-338.

54. Tucker ON, Dannenberg AJ, Yang EK, Zhang F, Teng L, et al. (1999) Cyclooxygenase-2 expression is up-regulated in human pancreatic cancer. Cancer Res 59: 987-990.

55. Schlosser W, Schlosser S, Ramadani M, Gansauge F, Gansauge S, et al. (2002) Cyclooxygenase-2 is overexpressed in chronic pancreatitis. Pancreas 25: 26-30.

56. Okami J, Yamamoto H, Fujiwara Y, Tsujie M, Kondo M, et al. (1999) Overexpression of cyclooxygenase-2 in carcinoma of the pancreas. Clin Cancer Res 5: 2018-2024.

57. Albazaz R, Verbeke CS, Rahman SH, McMahon MJ (2005) Cyclooxygenase-2 expression associated with severity of PanIN lesions: a possible link between chronic pancreatitis and pancreatic cancer. Pancreatology 5: 361-369.

58. Wang HX, Chen QK (2003) [Expression and significance of cyclooxygenase-2 in human pancreatic carcinomas]. Ai Zheng 22: 649-652.

59. Franco L, Doria D, Bertazzoni E, Benini A, Bassi C (2004) Increased expression of inducible nitric oxide synthase and cyclooxygenase-2 in pancreatic cancer. Prostaglandins Other Lipid Mediat 73: 51-58.

60. Kang SY, Lee JJ, Lee WI (2007) [Clinical significance of serum osteopontin in patients with multiple myeloma.]. Korean J Lab Med 27: 400-405.

61. Koopmann J, Rosenzweig CN, Zhang Z, Canto MI, Brown DA, et al. (2006) Serum markers in patients with resectable pancreatic adenocarcinoma: macrophage inhibitory cytokine 1 versus CA19-9. Clin Cancer Res 12: 442-446.

62. Molina H, Bunkenborg J, Reddy GH, Muthusamy B, Scheel PJ, et al. (2005) A proteomic analysis of human hemodialysis fluid. Mol Cell Proteomics 4: 637-650.

63. Christensen B, Petersen TE, Sorensen ES (2008) Post-translational modification and proteolytic processing of urinary osteopontin. Biochem J 411: 53-61.

64. Adachi J, Kumar C, Zhang Y, Olsen JV, Mann M (2006) The human urinary proteome contains more than 1500 proteins, including a large proportion of membrane proteins. Genome Biol 7: R80.

65. Zougman A, Pilch B, Podtelejnikov A, Kiehntopf M, Schnabel C, et al. (2008) Integrated analysis of the cerebrospinal fluid peptidome and proteome. J Proteome Res 7: 386-399.

66. Rogers CD, Fukushima N, Sato N, Shi C, Prasad N, et al. (2006) Differentiating pancreatic lesions by microarray and QPCR analysis of pancreatic juice RNAs. Cancer Biol Ther 5: 1383-1389.

67. Fukushima N, Koopmann J, Sato N, Prasad N, Carvalho R, et al. (2005) Gene expression alterations in the non-neoplastic parenchyma adjacent to infiltrating pancreatic ductal adenocarcinoma. Mod Pathol 18: 779-787.

68. Jin G, Hu XG, Ying K, Tang Y, Liu R, et al. (2005) Discovery and analysis of pancreatic adenocarcinoma genes using cDNA microarrays. World J Gastroenterol 11: 6543-6548.

69. Kolb A, Kleeff J, Guweidhi A, Esposito I, Giese NA, et al. (2005) Osteopontin influences the invasiveness of pancreatic cancer cells and is increased in neoplastic and inflammatory conditions. Cancer Biol Ther 4: 740-746.

70. Koopmann J, Fedarko NS, Jain A, Maitra A, Iacobuzio-Donahue C, et al. (2004) Evaluation of osteopontin as biomarker for pancreatic adenocarcinoma. Cancer Epidemiol Biomarkers Prev 13: 487-491.

71. Coppola D, Szabo M, Boulware D, Muraca P, Alsarraj M, et al. (2004) Correlation of osteopontin protein expression and pathological stage across a wide variety of tumor histologies. Clin Cancer Res 10: 184-190.

72. Sonderbye L, Meehan S, Palsson R, Ahsan N, Ladefoged J, et al. (1998) Immunohistochemical study of actin binding protein (p55) in the human kidney. Transplantation 65: 1004-1008.

73. Lin XH, Grako KA, Burg MA, Stallcup WB (1996) NG2 proteoglycan and the actin-binding protein fascin define separate populations of actin-containing filopodia and lamellipodia during cell spreading and migration. Mol Biol Cell 7: 1977-1993.

74. Tsai WC, Chao YC, Sheu LF, Chang JL, Nieh S, et al. (2007) Overexpression of fascin-1 in advanced colorectal adenocarcinoma: tissue microarray analysis of immunostaining scores with clinicopathological parameters. Dis Markers 23: 153-160.

75. Tsai WC, Chao YC, Sheu LF, Lin YF, Nieh S, et al. (2007) EMMPRIN and fascin overexpression associated with clinicopathologic parameters of pancreatobiliary adenocarcinoma in Chinese people. Apmis 115: 929-938.

76. Logsdon CD, Simeone DM, Binkley C, Arumugam T, Greenson JK, et al. (2003) Molecular profiling of pancreatic adenocarcinoma and chronic pancreatitis identifies multiple genes differentially regulated in pancreatic cancer. Cancer Res 63: 2649-2657.

77. Lu Z, Hu L, Evers S, Chen J, Shen Y (2004) Differential expression profiling of human pancreatic adenocarcinoma and healthy pancreatic tissue. Proteomics 4: 3975-3988.

78. Crnogorac-Jurcevic T, Missiaglia E, Blaveri E, Gangeswaran R, Jones M, et al. (2003) Molecular alterations in pancreatic carcinoma: expression profiling shows that dysregulated expression of S100 genes is highly prevalent. J Pathol 201: 63-74.

79. Buchholz M, Braun M, Heidenblut A, Kestler HA, Kloppel G, et al. (2005) Transcriptome analysis of microdissected pancreatic intraepithelial neoplastic lesions. Oncogene 24: 6626-6636.

80. Shekouh AR, Thompson CC, Prime W, Campbell F, Hamlett J, et al. (2003) Application of laser capture microdissection combined with two-dimensional electrophoresis for the discovery of differentially regulated proteins in pancreatic ductal adenocarcinoma. Proteomics 3: 1988-2001.

81. Ohuchida K, Mizumoto K, Ishikawa N, Fujii K, Konomi H, et al. (2005) The role of S100A6 in pancreatic cancer development and its clinical implication as a diagnostic marker and therapeutic target. Clin Cancer Res 11: 7785-7793.

82. Sager R, Sheng S, Pemberton P, Hendrix MJ (1997) Maspin. A tumor suppressing serpin. Adv Exp Med Biol 425: 77-88.

83. Pfeffer F, Koczan D, Adam U, Benz S, von Dobschuetz E, et al. (2004) Expression of connexin26 in islets of Langerhans is associated with impaired glucose tolerance in patients with pancreatic adenocarcinoma. Pancreas 29: 284-290.

84. Maass N, Hojo T, Ueding M, Luttges J, Kloppel G, et al. (2001) Expression of the tumor suppressor gene Maspin in human pancreatic cancers. Clin Cancer Res 7: 812-817.

85. Cao D, Zhang Q, Wu LS, Salaria SN, Winter JW, et al. (2007) Prognostic significance of maspin in pancreatic ductal adenocarcinoma: tissue microarray analysis of 223 surgically resected cases. Mod Pathol 20: 570-578.

86. Lim YJ, Lee JK, Jang WY, Song SY, Lee KT, et al. (2004) Prognostic significance of maspin in pancreatic ductal adenocarcinoma. Korean J Intern Med 19: 15-18.

87. Ohike N, Maass N, Mundhenke C, Biallek M, Zhang M, et al. (2003) Clinicopathological significance and molecular regulation of maspin expression in ductal adenocarcinoma of the pancreas. Cancer Lett 199: 193-200.

88. Kristiansen TZ, Bunkenborg J, Gronborg M, Molina H, Thuluvath PJ, et al. (2004) A proteomic analysis of human bile. Mol Cell Proteomics 3: 715-728.

89. Hibi Y, Fukushima N, Tsuchida A, Sofuni A, Itoi T, et al. (2007) Pancreatic juice cytology and subclassification of intraductal papillary mucinous neoplasms of the pancreas. Pancreas 34: 197-204.

90. Hovenberg HW, Davies JR, Herrmann A, Linden CJ, Carlstedt I (1996) MUC5AC, but not MUC2, is a prominent mucin in respiratory secretions. Glycoconj J 13: 839-847.

91. Luttges J, Feyerabend B, Buchelt T, Pacena M, Kloppel G (2002) The mucin profile of noninvasive and invasive mucinous cystic neoplasms of the pancreas. Am J Surg Pathol 26: 466-471.

92. Maitra A, Adsay NV, Argani P, Iacobuzio-Donahue C, De Marzo A, et al. (2003) Multicomponent analysis of the pancreatic adenocarcinoma progression model using a pancreatic intraepithelial neoplasia tissue microarray. Mod Pathol 16: 902-912.

93. Hanski C, Hofmeier M, Schmitt-Graff A, Riede E, Hanski ML, et al. (1997) Overexpression or ectopic expression of MUC2 is the common property of mucinous carcinomas of the colon, pancreas, breast, and ovary. J Pathol 182: 385-391.

94. Christensen B, Petersen TE, Sorensen ES (2007) Posttranslational modification and proteolytic processing of urinary osteopontin. Biochem J.

95. Missiaglia E, Blaveri E, Terris B, Wang YH, Costello E, et al. (2004) Analysis of gene expression in cancer cell lines identifies candidate markers for pancreatic tumorigenesis and metastasis. Int J Cancer 112: 100-112.

96. Chen R, Pan S, Yi EC, Donohoe S, Bronner MP, et al. (2006) Quantitative proteomic profiling of pancreatic cancer juice. Proteomics 6: 3871-3879.

97. Huhtala ML, Pesonen K, Kalkkinen N, Stenman UH (1982) Purification and characterization of a tumor-associated trypsin inhibitor from the urine of a patient with ovarian cancer. J Biol Chem 257: 13713-13716.

98. Freeman TC, Playford RJ, Quinn C, Beardshall K, Poulter L, et al. (1990) Pancreatic secretory trypsin inhibitor in gastrointestinal mucosa and gastric juice. Gut 31: 1318-1323.

99. Chen R, Pan S, Cooke K, Moyes KW, Bronner MP, et al. (2007) Comparison of pancreas juice proteins from cancer versus pancreatitis using quantitative proteomic analysis. Pancreas 34: 70-79.

100. Cristaudo A, Foddis R, Vivaldi A, Guglielmi G, Dipalma N, et al. (2007) Clinical significance of serum mesothelin in patients with mesothelioma and lung cancer. Clin Cancer Res 13: 5076-5081.

101. Creaney J, van Bruggen I, Hof M, Segal A, Musk AW, et al. (2007) Combined CA125 and mesothelin levels for the diagnosis of malignant mesothelioma. Chest 132: 1239-1246.

102. Hassan R, Remaley AT, Sampson ML, Zhang J, Cox DD, et al. (2006) Detection and quantitation of serum mesothelin, a tumor marker for patients with mesothelioma and ovarian cancer. Clin Cancer Res 12: 447-453.

103. Onda M, Nagata S, Ho M, Bera TK, Hassan R, et al. (2006) Megakaryocyte potentiation factor cleaved from mesothelin precursor is a useful tumor marker in the serum of patients with mesothelioma. Clin Cancer Res 12: 4225-4231.

104. Badgwell D, Lu Z, Cole L, Fritsche H, Atkinson EN, et al. (2007) Urinary mesothelin provides greater sensitivity for early stage ovarian cancer than serum mesothelin, urinary hCG free beta subunit and urinary hCG beta core fragment. Gynecol Oncol 106: 490-497.

105. Sato N, Fukushima N, Maitra A, Iacobuzio-Donahue CA, van Heek NT, et al. (2004) Gene expression profiling identifies genes associated with invasive intraductal papillary mucinous neoplasms of the pancreas. Am J Pathol 164: 903-914.

106. Argani P, Iacobuzio-Donahue C, Ryu B, Rosty C, Goggins M, et al. (2001) Mesothelin is overexpressed in the vast majority of ductal adenocarcinomas of the pancreas: identification of a new pancreatic cancer marker by serial analysis of gene expression (SAGE). Clin Cancer Res 7: 3862-3868.

107. Watanabe H, Okada G, Ohtsubo K, Yamaguchi Y, Mouri H, et al. (2005) Expression of mesothelin mRNA in pure pancreatic juice from patients with pancreatic carcinoma, intraductal papillary mucinous neoplasm of the pancreas, and chronic pancreatitis. Pancreas 30: 349-354.

108. Hassan R, Laszik ZG, Lerner M, Raffeld M, Postier R, et al. (2005) Mesothelin is overexpressed in pancreaticobiliary adenocarcinomas but not in normal pancreas and chronic pancreatitis. Am J Clin Pathol 124: 838-845.

109. Lohr M, Trautmann B, Gottler M, Peters S, Zauner I, et al. (1996) Expression and function of receptors for extracellular matrix proteins in human ductal adenocarcinomas of the pancreas. Pancreas 12: 248-259.

110. Ordonez NG (2003) Application of mesothelin immunostaining in tumor diagnosis. Am J Surg Pathol 27: 1418-1428.

111. Keleg S, Kayed H, Jiang X, Penzel R, Giese T, et al. (2007) Adrenomedullin is induced by hypoxia and enhances pancreatic cancer cell invasion. Int J Cancer 121: 21-32.

112. Nakatsuka M, Habara T, Noguchi S, Konishi H, Kudo T (2003) Increased plasma adrenomedullin in women with recurrent pregnancy loss. Obstet Gynecol 102: 319-324.

113. Kitamura K, Ichiki Y, Tanaka M, Kawamoto M, Emura J, et al. (1994) Immunoreactive adrenomedullin in human plasma. FEBS Lett 341: 288-290.

114. Kapas S, Pahal K, Cruchley AT, Hagi-Pavli E, Hinson JP (2004) Expression of adrenomedullin and its receptors in human salivary tissue. J Dent Res 83: 333-337.

115. Ramachandran V, Arumugam T, Hwang RF, Greenson JK, Simeone DM, et al. (2007) Adrenomedullin is expressed in pancreatic cancer and stimulates cell proliferation and invasion in an autocrine manner via the adrenomedullin receptor, ADMR. Cancer Res 67: 2666-2675.

116. Pavel ME, Hoppe S, Papadopoulos T, Linder V, Mohr B, et al. (2006) Adrenomedullin is a novel marker of tumor progression in neuroendocrine carcinomas. Horm Metab Res 38: 112-118.

117. Ishikawa T, Chen J, Wang J, Okada F, Sugiyama T, et al. (2003) Adrenomedullin antagonist suppresses in vivo growth of human pancreatic cancer cells in SCID mice by suppressing angiogenesis. Oncogene 22: 1238-1242.

118. Gronbaek H, Vestergaard EM, Hey H, Nielsen JN, Nexo E (2006) Serum trefoil factors in patients with inflammatory bowel disease. Digestion 74: 33-39.

119. Miyashita S, Nomoto H, Konishi H, Hayashi K (1994) Estimation of pS2 protein level in human body fluids by a sensitive two-site enzyme immunoassay. Clin Chim Acta 228: 71-81.

120. Vestergaard EM, Brynskov J, Ejskjaer K, Clausen JT, Thim L, et al. (2004) Immunoassays of human trefoil factors 1 and 2: measured on serum from patients with inflammatory bowel disease. Scand J Clin Lab Invest 64: 146-156.

121. Chenard MP, Tomasetto C, Bellocq JP, Rio MC (2004) Urinary pS2/TFF1 levels in the management of hormonodependent breast carcinomas. Peptides 25: 737-743.

122. Fukushima N, Sato N, Prasad N, Leach SD, Hruban RH, et al. (2004) Characterization of gene expression in mucinous cystic neoplasms of the pancreas using oligonucleotide microarrays. Oncogene 23: 9042-9051.

123. Johnson SK, Dennis RA, Barone GW, Lamps LW, Haun RS (2006) Differential expression of insulin-like growth factor binding protein-5 in pancreatic adenocarcinomas: identification using DNA microarray. Mol Carcinog 45: 814-827.

124. Terris B, Blaveri E, Crnogorac-Jurcevic T, Jones M, Missiaglia E, et al. (2002) Characterization of gene expression profiles in intraductal papillary-mucinous tumors of the pancreas. Am J Pathol 160: 1745-1754.

125. Friess H, Ding J, Kleeff J, Fenkell L, Rosinski JA, et al. (2003) Microarray-based identification of differentially expressed growth- and metastasis-associated genes in pancreatic cancer. Cell Mol Life Sci 60: 1180-1199.

126. Prasad NB, Biankin AV, Fukushima N, Maitra A, Dhara S, et al. (2005) Gene expression profiles in pancreatic intraepithelial neoplasia reflect the effects of Hedgehog signaling on pancreatic ductal epithelial cells. Cancer Res 65: 1619-1626.

127. Axelsson L, Bergenfeldt M, Ohlsson K (1995) Studies of the release and turnover of a human neutrophil lipocalin. Scand J Clin Lab Invest 55: 577-588.

128. Blaser J, Triebel S, Tschesche H (1995) A sandwich enzyme immunoassay for the determination of neutrophil lipocalin in body fluids. Clin Chim Acta 235: 137-145.

129. Iacobuzio-Donahue CA, Maitra A, Olsen M, Lowe AW, van Heek NT, et al. (2003) Exploration of global gene expression patterns in pancreatic adenocarcinoma using cDNA microarrays. Am J Pathol 162: 1151-1162.

130. Argani P, Rosty C, Reiter RE, Wilentz RE, Murugesan SR, et al. (2001) Discovery of new markers of cancer through serial analysis of gene expression: prostate stem cell antigen is overexpressed in pancreatic adenocarcinoma. Cancer Res 61: 4320-4324.

131. Sasaki H, Yu CY, Dai M, Tam C, Loda M, et al. (2003) Elevated serum periostin levels in patients with bone metastases from breast but not lung cancer. Breast Cancer Res Treat 77: 245-252.

132. Sasaki H, Roberts J, Lykins D, Fujii Y, Auclair D, et al. (2002) Novel chemiluminescence assay for serum periostin levels in women with preeclampsia and in normotensive pregnant women. Am J Obstet Gynecol 186: 103-108.

133. Sasaki H, Dai M, Auclair D, Kaji M, Fukai I, et al. (2001) Serum level of the periostin, a homologue of an insect cell adhesion molecule, in thymoma patients. Cancer Lett 172: 37-42.

134. Baril P, Gangeswaran R, Mahon PC, Caulee K, Kocher HM, et al. (2007) Periostin promotes invasiveness and resistance of pancreatic cancer cells to hypoxia-induced cell death: role of the beta4 integrin and the PI3k pathway. Oncogene 26: 2082-2094.

135. Erkan M, Kleeff J, Gorbachevski A, Reiser C, Mitkus T, et al. (2007) Periostin creates a tumor-supportive microenvironment in the pancreas by sustaining fibrogenic stellate cell activity. Gastroenterology 132: 1447-1464.

136. Zhou W, Sokoll LJ, Bruzek DJ, Zhang L, Velculescu VE, et al. (1998) Identifying markers for pancreatic cancer by gene expression analysis. Cancer Epidemiol Biomarkers Prev 7: 109-112.

137. Yukawa N, Yoshikawa T, Akaike M, Sugimasa Y, Rino Y, et al. (2007) Impact of Plasma Tissue Inhibitor of Matrix Metalloproteinase-1 on Long-Term Survival in Patients with Colorectal Cancer. Oncology 72: 205-208.

138. Sorensen NM, Bystrom P, Christensen IJ, Berglund A, Nielsen HJ, et al. (2007) TIMP-1 is significantly associated with objective response and survival in metastatic colorectal cancer patients receiving combination of irinotecan, 5-fluorouracil, and folinic acid. Clin Cancer Res 13: 4117-4122.

139. Caspersen MB, Sorensen NM, Schrohl AS, Iversen P, Nielsen HJ, et al. (2007) Investigation of tissue inhibitor of metalloproteinases 1 in plasma from colorectal cancer patients and blood donors by surface-enhanced laser desorption/ionization time-of-flight mass spectrometry. Int J Biol Markers 22: 89-94.

140. Emingil G, Tervahartiala T, Mantyla P, Maatta M, Sorsa T, et al. (2006) Gingival crevicular fluid matrix metalloproteinase (MMP)-7, extracellular MMP inducer, and tissue inhibitor of MMP-1 levels in periodontal disease. J Periodontol 77: 2040-2050.

141. Yang YH, Deng H, Li WM, Zhang QY, Hu XT, et al. (2008) Identification of matrix metalloproteinase 11 as a predictive tumor marker in serum based on gene expression profiling. Clin Cancer Res 14: 74-81.

142. Tirumalai RS, Chan KC, Prieto DA, Issaq HJ, Conrads TP, et al. (2003) Characterization of the low molecular weight human serum proteome. Mol Cell Proteomics 2: 1096-1103.

143. von Marschall Z, Riecken EO, Rosewicz S (1998) Stromelysin 3 is overexpressed in human pancreatic carcinoma and regulated by retinoic acid in pancreatic carcinoma cell lines. Gut 43: 692-698.

144. Rosty C, Christa L, Kuzdzal S, Baldwin WM, Zahurak ML, et al. (2002) Identification of hepatocarcinoma-intestine-pancreas/pancreatitis-associated protein I as a biomarker for pancreatic ductal adenocarcinoma by protein biochip technology. Cancer Res 62: 1868-1875.

145. Motoo Y, Watanabe H, Yamaguchi Y, Xie MJ, Mouri H, et al. (2001) Pancreatitis-associated protein levels in pancreatic juice from patients with pancreatic diseases. Pancreatology 1: 43-47.

146. Xie MJ, Motoo Y, Iovanna JL, Su SB, Ohtsubo K, et al. (2003) Overexpression of pancreatitis-associated protein (PAP) in human pancreatic ductal adenocarcinoma. Dig Dis Sci 48: 459-464.

147. Cerwenka H, Aigner R, Bacher H, Werkgartner G, el-Shabrawi A, et al. (2001) Pancreatitis-associated protein (PAP) in patients with pancreatic cancer. Anticancer Res 21: 1471-1474.

148. Hlatky MA, Ashley E, Quertermous T, Boothroyd DB, Ridker P, et al. (2007) Matrix metalloproteinase circulating levels, genetic polymorphisms, and susceptibility to acute myocardial infarction among patients with coronary artery disease. Am Heart J 154: 1043-1051.

149. Guo CB, Wang S, Deng C, Zhang DL, Wang FL, et al. (2007) Relationship between matrix metalloproteinase 2 and lung cancer progression. Mol Diagn Ther 11: 183-192.

150. Wilson S, Wakelam MJ, Hobbs RF, Ryan AV, Dunn JA, et al. (2006) Evaluation of the accuracy of serum MMP-9 as a test for colorectal cancer in a primary care population. BMC Cancer 6: 258.

151. Segara D, Biankin AV, Kench JG, Langusch CC, Dawson AC, et al. (2005) Expression of HOXB2, a retinoic acid signaling target in pancreatic cancer and pancreatic intraepithelial neoplasia. Clin Cancer Res 11: 3587-3596.

152. Wagner M, Kleeff J, Friess H, Buchler MW, Korc M (1999) Enhanced expression of the type II transforming growth factor-beta receptor is associated with decreased survival in human pancreatic cancer. Pancreas 19: 370-376.

153. Kleeff J, Friess H, Simon P, Susmallian S, Buchler P, et al. (1999) Overexpression of Smad2 and colocalization with TGF-beta1 in human pancreatic cancer. Dig Dis Sci 44: 1793-1802.

154. Gurevich LE (2003) Role of matrix metalloproteinases 2 and 9 in determination of invasive potential of pancreatic tumors. Bull Exp Biol Med 136: 494-498.

155. Harvey SR, Hurd TC, Markus G, Martinick MI, Penetrante RM, et al. (2003) Evaluation of urinary plasminogen activator, its receptor, matrix metalloproteinase-9, and von Willebrand factor in pancreatic cancer. Clin Cancer Res 9: 4935-4943.

156. Qian X, Rothman VL, Nicosia RF, Tuszynski GP (2001) Expression of thrombospondin-1 in human pancreatic adenocarcinomas: role in matrix metalloproteinase-9 production. Pathol Oncol Res 7: 251-259.

157. Scalabrini D, Fenoglio C, Scarpini E, De Riz M, Comi C, et al. (2007) Candidate gene analysis of SPARCL1 gene in patients with multiple sclerosis. Neurosci Lett 425: 173-176.

158. Esposito I, Kayed H, Keleg S, Giese T, Sage EH, et al. (2007) Tumor-suppressor function of SPARC-like protein 1/Hevin in pancreatic cancer. Neoplasia 9: 8-17.

159. Kayton ML, Costouros NG, Lorang D, Alexander HR, Hewitt SM, et al. (2003) Peak stimulated insulin secretion is associated with specific changes in gene expression profiles in sporadic insulinomas. Surgery 134: 982-987; discussion 987-988.

160. Ryu B, Jones J, Hollingsworth MA, Hruban RH, Kern SE (2001) Invasion-specific genes in malignancy: serial analysis of gene expression comparisons of primary and passaged cancers. Cancer Res 61: 1833-1838.

161. Bamrungphon W, Prempracha N, Bunchu N, Rangdaeng S, Sandhu T, et al. (2007) A new mucin antibody/enzyme-linked lectin-sandwich assay of serum MUC5AC mucin for the diagnosis of cholangiocarcinoma. Cancer Lett 247: 301-308.

162. Boonla C, Wongkham S, Sheehan JK, Wongkham C, Bhudhisawasdi V, et al. (2003) Prognostic value of serum MUC5AC mucin in patients with cholangiocarcinoma. Cancer 98: 1438-1443.

163. Kocer B, McKolanis J, Soran A (2006) Humoral immune response to MUC5AC in patients with colorectal polyps and colorectal carcinoma. BMC Gastroenterol 6: 4.

164. Grutzmann R, Foerder M, Alldinger I, Staub E, Brummendorf T, et al. (2003) Gene expression profiles of microdissected pancreatic ductal adenocarcinoma. Virchows Arch 443: 508-517.

165. Kanno A, Satoh K, Kimura K, Hirota M, Umino J, et al. (2006) The expression of MUC4 and MUC5AC is related to the biologic malignancy of intraductal papillary mucinous neoplasms of the pancreas. Pancreas 33: 391-396.

166. Ramsauer VP, Carraway CA, Salas PJ, Carraway KL (2003) Muc4/sialomucin complex, the intramembrane ErbB2 ligand, translocates ErbB2 to the apical surface in polarized epithelial cells. J Biol Chem 278: 30142-30147.

167. Duraisamy S, Ramasamy S, Kharbanda S, Kufe D (2006) Distinct evolution of the human carcinoma-associated transmembrane mucins, MUC1, MUC4 AND MUC16. Gene 373: 28-34.

168. Alameda F, Mejias-Luque R, Garrido M, de Bolos C (2007) Mucin genes (MUC2, MUC4, MUC5AC, and MUC6) detection in normal and pathological endometrial tissues. Int J Gynecol Pathol 26: 61-65.

169. Zhang J, Yasin M, Carraway CA, Carraway KL (2006) MUC4 expression and localization in gastrointestinal tract and skin of human embryos. Tissue Cell 38: 271-275.

170. Moniaux N, Escande F, Batra SK, Porchet N, Laine A, et al. (2000) Alternative splicing generates a family of putative secreted and membrane-associated MUC4 mucins. Eur J Biochem 267: 4536-4544.

171. Choudhury A, Moniaux N, Winpenny JP, Hollingsworth MA, Aubert JP, et al. (2000) Human MUC4 mucin cDNA and its variants in pancreatic carcinoma. J Biochem (Tokyo) 128: 233-243.

172. Spurr-Michaud S, Argueso P, Gipson I (2007) Assay of mucins in human tear fluid. Exp Eye Res 84: 939-950.

173. Liu B, Offner GD, Nunes DP, Oppenheim FG, Troxler RF (1998) MUC4 is a major component of salivary mucin MG1 secreted by the human submandibular gland. Biochem Biophys Res Commun 250: 757-761.

174. Singh AP, Chauhan SC, Andrianifahanana M, Moniaux N, Meza JL, et al. (2007) MUC4 expression is regulated by cystic fibrosis transmembrane conductance regulator in pancreatic adenocarcinoma cells via transcriptional and post-translational mechanisms. Oncogene 26: 30-41.

175. Li XH, Xiong JX, Wang CY (2005) [Clinical significance of the expression of MUC4 mRNA in peripheral blood mononuclear cells of pancreatic cancer patients]. Zhongguo Yi Xue Ke Xue Yuan Xue Bao 27: 624-627.

176. Choudhury A, Moniaux N, Ulrich AB, Schmied BM, Standop J, et al. (2004) MUC4 mucin expression in human pancreatic tumours is affected by organ environment: the possible role of TGFbeta2. Br J Cancer 90: 657-664.

177. Park HU, Kim JW, Kim GE, Bae HI, Crawley SC, et al. (2003) Aberrant expression of MUC3 and MUC4 membrane-associated mucins and sialyl Le(x) antigen in pancreatic intraepithelial neoplasia. Pancreas 26: e48-54.

178. Moniaux N, Varshney GC, Chauhan SC, Copin MC, Jain M, et al. (2004) Generation and characterization of anti-MUC4 monoclonal antibodies reactive with normal and cancer cells in humans. J Histochem Cytochem 52: 253-261.

179. Bhardwaj A, Marsh WL, Jr., Nash JW, Barbacioru CC, Jones S, et al. (2007) Double immunohistochemical staining with MUC4/p53 is useful in the distinction of pancreatic adenocarcinoma from chronic pancreatitis: a tissue microarray-based study. Arch Pathol Lab Med 131: 556-562.

180. Swartz MJ, Batra SK, Varshney GC, Hollingsworth MA, Yeo CJ, et al. (2002) MUC4 expression increases progressively in pancreatic intraepithelial neoplasia. Am J Clin Pathol 117: 791-796.

181. Cruz-Monserrate Z, Qiu S, Evers BM, O'Connor KL (2007) Upregulation and redistribution of integrin alpha6beta4 expression occurs at an early stage in pancreatic adenocarcinoma progression. Mod Pathol 20: 656-667.

182. Sawai H, Okada Y, Funahashi H, Matsuo Y, Takahashi H, et al. (2006) Interleukin-1alpha enhances the aggressive behavior of pancreatic cancer cells by regulating the alpha6beta1-integrin and urokinase plasminogen activator receptor expression. BMC Cell Biol 7: 8.

183. Gesierich S, Paret C, Hildebrand D, Weitz J, Zgraggen K, et al. (2005) Colocalization of the tetraspanins, CO-029 and CD151, with integrins in human pancreatic adenocarcinoma: impact on cell motility. Clin Cancer Res 11: 2840-2852.

184. Halatsch ME, Hirsch-Ernst KI, Kahl GF, Weinel RJ (1997) Increased expression of alpha6-integrin receptors and of mRNA encoding the putative 37 kDa laminin receptor precursor in pancreatic carcinoma. Cancer Lett 118: 7-11.

185. Min HY, Semnani R, Mizukami IF, Watt K, Todd RF, 3rd, et al. (1992) cDNA for Mo3, a monocyte activation antigen, encodes the human receptor for urokinase plasminogen activator. J Immunol 148: 3636-3642.

186. Mukhina S, Stepanova V, Traktouev D, Poliakov A, Beabealashvilly R, et al. (2000) The chemotactic action of urokinase on smooth muscle cells is dependent on its kringle domain. Characterization of interactions and contribution to chemotaxis. J Biol Chem 275: 16450-16458.

187. Casey JR, Petranka JG, Kottra J, Fleenor DE, Rosse WF (1994) The structure of the urokinase-type plasminogen activator receptor gene. Blood 84: 1151-1156.

188. Shin BK, Wang H, Yim AM, Le Naour F, Brichory F, et al. (2003) Global profiling of the cell surface proteome of cancer cells uncovers an abundance of proteins with chaperone function. J Biol Chem 278: 7607-7616.

189. Kasperska-Zajac A, Brzoza Z, Rogala B (2007) Blood urokinase plasminogen activator system in chronic urticaria. Arch Dermatol Res 298: 409-411.

190. Grebenchtchikov N, Maguire TM, Riisbro R, Geurts-Moespot A, O'Donovan N, et al. (2005) Measurement of plasminogen activator system components in plasma and tumor tissue extracts obtained from patients with breast cancer: an EORTC Receptor and Biomarker Group collaboration. Oncol Rep 14: 235-239.

191. Florquin S, van den Berg JG, Olszyna DP, Claessen N, Opal SM, et al. (2001) Release of urokinase plasminogen activator receptor during urosepsis and endotoxemia. Kidney Int 59: 2054-2061.

192. Shariat SF, Roehrborn CG, McConnell JD, Park S, Alam N, et al. (2007) Association of the circulating levels of the urokinase system of plasminogen activation with the presence of prostate cancer and invasion, progression, and metastasis. J Clin Oncol 25: 349-355.

193. Sporer B, Koedel U, Popp B, Paul R, Pfister HW (2005) Evaluation of cerebrospinal fluid uPA, PAI-1, and soluble uPAR levels in HIV-infected patients. J Neuroimmunol 163: 190-194.

194. Arimochi J, Ohashi-Kobayashi A, Maeda M (2007) Interaction of Mat-8 (FXYD-3) with Na+/K+-ATPase in colorectal cancer cells. Biol Pharm Bull 30: 648-654.

195. Kayed H, Kleeff J, Kolb A, Ketterer K, Keleg S, et al. (2006) FXYD3 is overexpressed in pancreatic ductal adenocarcinoma and influences pancreatic cancer cell growth. Int J Cancer 118: 43-54.

196. Morrison BW, Moorman JR, Kowdley GC, Kobayashi YM, Jones LR, et al. (1995) Mat-8, a novel phospholemman-like protein expressed in human breast tumors, induces a chloride conductance in Xenopus oocytes. J Biol Chem 270: 2176-2182.

197. Weskamp G, Kratzschmar J, Reid MS, Blobel CP (1996) MDC9, a widely expressed cellular disintegrin containing cytoplasmic SH3 ligand domains. J Cell Biol 132: 717-726.

198. Grutzmann R, Luttges J, Sipos B, Ammerpohl O, Dobrowolski F, et al. (2004) ADAM9 expression in pancreatic cancer is associated with tumour type and is a prognostic factor in ductal adenocarcinoma. Br J Cancer 90: 1053-1058.

199. Besleaga R, Montesinos-Rongen M, Perez-Tur J, Siebert R, Deckert M (2003) Expression of the LGI1 gene product in astrocytic gliomas: downregulation with malignant progression. Virchows Arch 443: 561-564.

200. Berditchevski F, Chang S, Bodorova J, Hemler ME (1997) Generation of monoclonal antibodies to integrin-associated proteins. Evidence that alpha3beta1 complexes with EMMPRIN/basigin/OX47/M6. J Biol Chem 272: 29174-29180.

201. Zhang W, Erkan M, Abiatari I, Giese NA, Felix K, et al. (2007) Expression of extracellular matrix metalloproteinase inducer (EMMPRIN/CD147) in pancreatic neoplasm and pancreatic stellate cells. Cancer Biol Ther 6: 218-227.

202. Li M, Zhai Q, Bharadwaj U, Wang H, Li F, et al. (2006) Cyclophilin A is overexpressed in human pancreatic cancer cells and stimulates cell proliferation through CD147. Cancer 106: 2284-2294.

203. Schneiderhan W, Diaz F, Fundel M, Zhou S, Siech M, et al. (2007) Pancreatic stellate cells are an important source of MMP-2 in human pancreatic cancer and accelerate tumor progression in a murine xenograft model and CAM assay. J Cell Sci 120: 512-519.

204. Riethdorf S, Reimers N, Assmann V, Kornfeld JW, Terracciano L, et al. (2006) High incidence of EMMPRIN expression in human tumors. Int J Cancer 119: 1800-1810.

205. Guignot J, Peiffer I, Bernet-Camard MF, Lublin DM, Carnoy C, et al. (2000) Recruitment of CD55 and CD66e brush border-associated glycosylphosphatidylinositol-anchored proteins by members of the Afa/Dr diffusely adhering family of Escherichia coli that infect the human polarized intestinal Caco-2/TC7 cells. Infect Immun 68: 3554-3563.

206. Screaton RA, DeMarte L, Draber P, Stanners CP (2000) The specificity for the differentiation blocking activity of carcinoembryonic antigen resides in its glycophosphatidyl-inositol anchor. J Cell Biol 150: 613-626.

207. Anderson NL, Polanski M, Pieper R, Gatlin T, Tirumalai RS, et al. (2004) The human plasma proteome: a nonredundant list developed by combination of four separate sources. Mol Cell Proteomics 3: 311-326.

208. Blumenthal RD, Leon E, Hansen HJ, Goldenberg DM (2007) Expression patterns of CEACAM5 and CEACAM6 in primary and metastatic cancers. BMC Cancer 7: 2.

209. Kristiansen G, Jacob J, Buckendahl AC, Grutzmann R, Alldinger I, et al. (2006) Peroxisome proliferator-activated receptor gamma is highly expressed in pancreatic cancer and is associated with shorter overall survival times. Clin Cancer Res 12: 6444-6451.

210. Ding Z, Issekutz TB, Downey GP, Waddell TK (2003) L-selectin stimulation enhances functional expression of surface CXCR4 in lymphocytes: implications for cellular activation during adhesion and migration. Blood 101: 4245-4252.

211. Babcock GJ, Farzan M, Sodroski J (2003) Ligand-independent dimerization of CXCR4, a principal HIV-1 coreceptor. J Biol Chem 278: 3378-3385.

212. Maitra A, Hansel DE, Argani P, Ashfaq R, Rahman A, et al. (2003) Global expression analysis of well-differentiated pancreatic endocrine neoplasms using oligonucleotide microarrays. Clin Cancer Res 9: 5988-5995.

213. Man XY, Yang XH, Cai SQ, Yao YG, Zheng M (2006) Immunolocalization and expression of vascular endothelial growth factor receptors (VEGFRs) and neuropilins (NRPs) on keratinocytes in human epidermis. Mol Med 12: 127-136.

214. Ghez D, Lepelletier Y, Lambert S, Fourneau JM, Blot V, et al. (2006) Neuropilin-1 is involved in human T-cell lymphotropic virus type 1 entry. J Virol 80: 6844-6854.

215. Gagnon ML, Bielenberg DR, Gechtman Z, Miao HQ, Takashima S, et al. (2000) Identification of a natural soluble neuropilin-1 that binds vascular endothelial growth factor: In vivo expression and antitumor activity. Proc Natl Acad Sci U S A 97: 2573-2578.

216. Parikh AA, Liu WB, Fan F, Stoeltzing O, Reinmuth N, et al. (2003) Expression and regulation of the novel vascular endothelial growth factor receptor neuropilin-1 by epidermal growth factor in human pancreatic carcinoma. Cancer 98: 720-729.

217. Fukahi K, Fukasawa M, Neufeld G, Itakura J, Korc M (2004) Aberrant expression of neuropilin-1 and -2 in human pancreatic cancer cells. Clin Cancer Res 10: 581-590.

218. Muller MW, Giese NA, Swiercz JM, Ceyhan GO, Esposito I, et al. (2007) Association of axon guidance factor semaphorin 3A with poor outcome in pancreatic cancer. Int J Cancer 121: 2421-2433.

219. Hansel DE, Wilentz RE, Yeo CJ, Schulick RD, Montgomery E, et al. (2004) Expression of neuropilin-1 in high-grade dysplasia, invasive cancer, and metastases of the human gastrointestinal tract. Am J Surg Pathol 28: 347-356.

220. Morrissette JD, Colliton RP, Spinner NB (2001) Defective intracellular transport and processing of JAG1 missense mutations in Alagille syndrome. Hum Mol Genet 10: 405-413.

221. Aho S (2004) Soluble form of Jagged1: unique product of epithelial keratinocytes and a regulator of keratinocyte differentiation. J Cell Biochem 92: 1271-1281.

222. Buchler P, Gazdhar A, Schubert M, Giese N, Reber HA, et al. (2005) The Notch signaling pathway is related to neurovascular progression of pancreatic cancer. Ann Surg 242: 791-800, discussion 800-791.

223. Ueda J, Semba S, Chiba H, Sawada N, Seo Y, et al. (2007) Heterogeneous expression of claudin-4 in human colorectal cancer: decreased claudin-4 expression at the invasive front correlates cancer invasion and metastasis. Pathobiology 74: 32-41.

224. Nichols LS, Ashfaq R, Iacobuzio-Donahue CA (2004) Claudin 4 protein expression in primary and metastatic pancreatic cancer: support for use as a therapeutic target. Am J Clin Pathol 121: 226-230.

225. Michl P, Buchholz M, Rolke M, Kunsch S, Lohr M, et al. (2001) Claudin-4: a new target for pancreatic cancer treatment using Clostridium perfringens enterotoxin. Gastroenterology 121: 678-684.

226. Foss CA, Fox JJ, Feldmann G, Maitra A, Iacobuzio-Donohue C, et al. (2007) Radiolabeled anti-claudin 4 and anti-prostate stem cell antigen: initial imaging in experimental models of pancreatic cancer. Mol Imaging 6: 131-139.

227. Sitek B, Luttges J, Marcus K, Kloppel G, Schmiegel W, et al. (2005) Application of fluorescence difference gel electrophoresis saturation labelling for the analysis of microdissected precursor lesions of pancreatic ductal adenocarcinoma. Proteomics 5: 2665-2679.

228. Hindmarsh EJ, Marks RM (1998) Decay-accelerating factor is a component of subendothelial extracellular matrix in vitro, and is augmented by activation of endothelial protein kinase C. Eur J Immunol 28: 1052-1062.

229. Taylor CT, Johnson PM (1996) Complement-binding proteins are strongly expressed by human preimplantation blastocysts and cumulus cells as well as gametes. Mol Hum Reprod 2: 52-59.

230. Lowe AW, Olsen M, Hao Y, Lee SP, Taek Lee K, et al. (2007) Gene expression patterns in pancreatic tumors, cells and tissues. PLoS ONE 2: e323.

231. Ramachandran P, Boontheung P, Xie Y, Sondej M, Wong DT, et al. (2006) Identification of N-linked glycoproteins in human saliva by glycoprotein capture and mass spectrometry. J Proteome Res 5: 1493-1503.

232. Kuroki M, Kuroki M, Moore GE, Ichiki S, Matsuoka Y (1988) The molecular heterogeneity of nonspecific cross-reacting antigen synthesized by tumor cells and granulocytes. Jpn J Cancer Res 79: 82-90.

233. Barnett T, Goebel SJ, Nothdurft MA, Elting JJ (1988) Carcinoembryonic antigen family: characterization of cDNAs coding for NCA and CEA and suggestion of nonrandom sequence variation in their conserved loop-domains. Genomics 3: 59-66.

234. Duxbury MS, Matros E, Clancy T, Bailey G, Doff M, et al. (2005) CEACAM6 is a novel biomarker in pancreatic adenocarcinoma and PanIN lesions. Ann Surg 241: 491-496.

235. Gress TM, Wallrapp C, Frohme M, Muller-Pillasch F, Lacher U, et al. (1997) Identification of genes with specific expression in pancreatic cancer by cDNA representational difference analysis. Genes Chromosomes Cancer 19: 97-103.

236. Ryu B, Jones J, Blades NJ, Parmigiani G, Hollingsworth MA, et al. (2002) Relationships and differentially expressed genes among pancreatic cancers examined by large-scale serial analysis of gene expression. Cancer Res 62: 819-826.

237. Katahira J, Inoue N, Horiguchi Y, Matsuda M, Sugimoto N (1997) Molecular cloning and functional characterization of the receptor for Clostridium perfringens enterotoxin. J Cell Biol 136: 1239-1247.

238. Azuma T, Hirai M, Ito S, Yamamoto K, Taggart RT, et al. (1996) Expression of cathepsin E in pancreas: a possible tumor marker for pancreas, a preliminary report. Int J Cancer 67: 492-497.

239. Uno K, Azuma T, Nakajima M, Yasuda K, Hayakumo T, et al. (2000) Clinical significance of cathepsin E in pancreatic juice in the diagnosis of pancreatic ductal adenocarcinoma. J Gastroenterol Hepatol 15: 1333-1338.

240. Takeda-Ezaki M, Yamamoto K (1993) Isolation and biochemical characterization of procathepsin E from human erythrocyte membranes. Arch Biochem Biophys 304: 352-358.

241. Forthoffer N, Gomez-Diaz C, Bello RI, Buron MI, Martin SF, et al. (2002) A novel plasma membrane quinone reductase and NAD(P)H:quinone oxidoreductase 1 are upregulated by serum withdrawal in human promyelocytic HL-60 cells. J Bioenerg Biomembr 34: 209-219.

242. Lewis AM, Ough M, Hinkhouse MM, Tsao MS, Oberley LW, et al. (2005) Targeting NAD(P)H:quinone oxidoreductase (NQO1) in pancreatic cancer. Mol Carcinog 43: 215-224.

243. Berberat PO, Friess H, Wang L, Zhu Z, Bley T, et al. (2001) Comparative analysis of galectins in primary tumors and tumor metastasis in human pancreatic cancer. J Histochem Cytochem 49: 539-549.

244. Shen J, Person MD, Zhu J, Abbruzzese JL, Li D (2004) Protein expression profiles in pancreatic adenocarcinoma compared with normal pancreatic tissue and tissue affected by pancreatitis as detected by two-dimensional gel electrophoresis and mass spectrometry. Cancer Res 64: 9018-9026.

245. Niimi T, Nagashima K, Ward JM, Minoo P, Zimonjic DB, et al. (2001) claudin-18, a novel downstream target gene for the T/EBP/NKX2.1 homeodomain transcription factor, encodes lung- and stomach-specific isoforms through alternative splicing. Mol Cell Biol 21: 7380-7390.

246. Karanjawala ZE, Illei PB, Ashfaq R, Infante JR, Murphy K, et al. (2008) New markers of pancreatic cancer identified through differential gene expression analyses: claudin 18 and annexin A8. Am J Surg Pathol 32: 188-196.

247. Drake TA, Morrissey JH, Edgington TS (1989) Selective cellular expression of tissue factor in human tissues. Implications for disorders of hemostasis and thrombosis. Am J Pathol 134: 1087-1097.

248. Carson SD, Henry WM, Shows TB (1985) Tissue factor gene localized to human chromosome 1 (1pter----1p21). Science 229: 991-993.

249. Chen VM, Ahamed J, Versteeg HH, Berndt MC, Ruf W, et al. (2006) Evidence for activation of tissue factor by an allosteric disulfide bond. Biochemistry 45: 12020-12028.

250. Khorana AA, Ahrendt SA, Ryan CK, Francis CW, Hruban RH, et al. (2007) Tissue factor expression, angiogenesis, and thrombosis in pancreatic cancer. Clin Cancer Res 13: 2870-2875.

251. Haas SL, Jesnowski R, Steiner M, Hummel F, Ringel J, et al. (2006) Expression of tissue factor in pancreatic adenocarcinoma is associated with activation of coagulation. World J Gastroenterol 12: 4843-4849.

252. Yamaguchi H, Inoue T, Eguchi T, Miyasaka Y, Ohuchida K, et al. (2007) Fascin overexpression in intraductal papillary mucinous neoplasms (adenomas, borderline neoplasms, and carcinomas) of the pancreas, correlated with increased histological grade. Mod Pathol 20: 552-561.

253. Maitra A, Iacobuzio-Donahue C, Rahman A, Sohn TA, Argani P, et al. (2002) Immunohistochemical validation of a novel epithelial and a novel stromal marker of pancreatic ductal adenocarcinoma identified by global expression microarrays: sea urchin fascin homolog and heat shock protein 47. Am J Clin Pathol 118: 52-59.

254. Sakamoto O, Iwama A, Amitani R, Takehara T, Yamaguchi N, et al. (1997) Role of macrophage-stimulating protein and its receptor, RON tyrosine kinase, in ciliary motility. J Clin Invest 99: 701-709.

255. Gaudino G, Follenzi A, Naldini L, Collesi C, Santoro M, et al. (1994) RON is a heterodimeric tyrosine kinase receptor activated by the HGF homologue MSP. Embo J 13: 3524-3532.

256. Wang MH, Ronsin C, Gesnel MC, Coupey L, Skeel A, et al. (1994) Identification of the ron gene product as the receptor for the human macrophage stimulating protein. Science 266: 117-119.

257. Thomas RM, Toney K, Fenoglio-Preiser C, Revelo-Penafiel MP, Hingorani SR, et al. (2007) The RON receptor tyrosine kinase mediates oncogenic phenotypes in pancreatic cancer cells and is increasingly expressed during pancreatic cancer progression. Cancer Res 67: 6075-6082.

258. Camp ER, Yang A, Gray MJ, Fan F, Hamilton SR, et al. (2007) Tyrosine kinase receptor RON in human pancreatic cancer: expression, function, and validation as a target. Cancer 109: 1030-1039.

259. Moniaux N, Junker WM, Singh AP, Jones AM, Batra SK (2006) Characterization of human mucin MUC17. Complete coding sequence and organization. J Biol Chem 281: 23676-23685.

260. Moniaux N, Nollet S, Porchet N, Degand P, Laine A, et al. (1999) Complete sequence of the human mucin MUC4: a putative cell membrane-associated mucin. Biochem J 338 (Pt 2): 325-333.

261. Hu YP, Haq B, Carraway KL, Savaraj N, Lampidis TJ (2003) Multidrug resistance correlates with overexpression of Muc4 but inversely with P-glycoprotein and multidrug resistance related protein in transfected human melanoma cells. Biochem Pharmacol 65: 1419-1425.

262. Andrianifahanana M, Chauhan SC, Choudhury A, Moniaux N, Brand RE, et al. (2006) MUC4-expressing pancreatic adenocarcinomas show elevated levels of both T1 and T2 cytokines: potential pathobiologic implications. Am J Gastroenterol 101: 2319-2329.

263. Kosmahl M, Wagner J, Peters K, Sipos B, Kloppel G (2004) Serous cystic neoplasms of the pancreas: an immunohistochemical analysis revealing alpha-inhibin, neuron-specific enolase, and MUC6 as new markers. Am J Surg Pathol 28: 339-346.

264. Bartman AE, Buisine MP, Aubert JP, Niehans GA, Toribara NW, et al. (1998) The MUC6 secretory mucin gene is expressed in a wide variety of epithelial tissues. J Pathol 186: 398-405.

265. Koltzscher M, Neumann C, Konig S, Gerke V (2003) Ca2+-dependent binding and activation of dormant ezrin by dimeric S100P. Mol Biol Cell 14: 2372-2384.

266. Ohuchida K, Mizumoto K, Egami T, Yamaguchi H, Fujii K, et al. (2006) S100P is an early developmental marker of pancreatic carcinogenesis. Clin Cancer Res 12: 5411-5416.

267. Dowen SE, Crnogorac-Jurcevic T, Gangeswaran R, Hansen M, Eloranta JJ, et al. (2005) Expression of S100P and its novel binding partner S100PBPR in early pancreatic cancer. Am J Pathol 166: 81-92.

268. Burgess JA, Lescuyer P, Hainard A, Burkhard PR, Turck N, et al. (2006) Identification of brain cell death associated proteins in human post-mortem cerebrospinal fluid. J Proteome Res 5: 1674-1681.

269. Kilani RT, Maksymowych WP, Aitken A, Boire G, St-Pierre Y, et al. (2007) Detection of high levels of 2 specific isoforms of 14-3-3 proteins in synovial fluid from patients with joint inflammation. J Rheumatol 34: 1650-1657.

270. Hustinx SR, Fukushima N, Zahurak ML, Riall TS, Maitra A, et al. (2005) Expression and prognostic significance of 14-3-3sigma and ERM family protein expression in periampullary neoplasms. Cancer Biol Ther 4: 596-601.

271. Guweidhi A, Kleeff J, Giese N, El Fitori J, Ketterer K, et al. (2004) Enhanced expression of 14-3-3sigma in pancreatic cancer and its role in cell cycle regulation and apoptosis. Carcinogenesis 25: 1575-1585.

272. Yukawa N, Yoshikawa T, Akaike M, Sugimasa Y, Rino Y, et al. (2007) Impact of plasma tissue inhibitor of matrix metalloproteinase-1 on long-term survival in patients with colorectal cancer. Oncology 72: 205-208.

273. Baumgart E, Lenk SV, Loening SA, Jung K (2002) Tissue inhibitors of metalloproteinases 1 and 2 in human seminal plasma and their association with spermatozoa. Int J Androl 25: 369-371.

274. Crnogorac-Jurcevic T, Efthimiou E, Capelli P, Blaveri E, Baron A, et al. (2001) Gene expression profiles of pancreatic cancer and stromal desmoplasia. Oncogene 20: 7437-7446.

275. Miyake H, Hara I, Yamanaka K, Gohji K, Arakawa S, et al. (1999) Elevation of serum levels of urokinase-type plasminogen activator and its receptor is associated with disease progression and prognosis in patients with prostate cancer. Prostate 39: 123-129.

276. Ueshima S, Matsumoto H, Izaki S, Mitsui Y, Fukao H, et al. (1999) Co-localization of urokinase and its receptor on established human umbilical vein endothelial cell. Cell Struct Funct 24: 71-78.

277. Nielsen A, Scarlett CJ, Samra JS, Gill A, Li Y, et al. (2005) Significant overexpression of urokinase-type plasminogen activator in pancreatic adenocarcinoma using real-time quantitative reverse transcription polymerase chain reaction. J Gastroenterol Hepatol 20: 256-263.

278. Wang W, Abbruzzese JL, Evans DB, Chiao PJ (1999) Overexpression of urokinase-type plasminogen activator in pancreatic adenocarcinoma is regulated by constitutively activated RelA. Oncogene 18: 4554-4563.

279. Boyd AW, Ward LD, Wicks IP, Simpson RJ, Salvaris E, et al. (1992) Isolation and characterization of a novel receptor-type protein tyrosine kinase (hek) from a human pre-B cell line. J Biol Chem 267: 3262-3267.

280. Binkley CE, Zhang L, Greenson JK, Giordano TJ, Kuick R, et al. (2004) The molecular basis of pancreatic fibrosis: common stromal gene expression in chronic pancreatitis and pancreatic adenocarcinoma. Pancreas 29: 254-263.

281. Yesudian PD, Klafkowski J, Parslew R, Gould D, Lloyd D, et al. (2007) Tufted angioma-associated Kasabach-Merritt syndrome treated with embolization and vincristine. Plast Reconstr Surg 119: 1392-1393.

282. Bellone G, Smirne C, Mauri FA, Tonel E, Carbone A, et al. (2006) Cytokine expression profile in human pancreatic carcinoma cells and in surgical specimens: implications for survival. Cancer Immunol Immunother 55: 684-698.

283. Goetzl L, Evans T, Rivers J, Suresh MS, Lieberman E (2002) Elevated maternal and fetal serum interleukin-6 levels are associated with epidural fever. Am J Obstet Gynecol 187: 834-838.

284. Morelli SS, Keegan DA, Krey LC, Katz J, Liu M, et al. (2008) Early serum interleukin-8 evaluation may prove useful in localizing abnormally implanted human gestations after in vitro fertilization. Fertil Steril.

285. Yang SQ, Xu JG (2008) [Effect of glutamine on serum interleukin-8 and tumor necrosis factor-alpha levels in patients with severe pancreatitis.]. Nan Fang Yi Ke Da Xue Xue Bao 28: 129-131.

286. Yasui T, Uemura H, Yamada M, Matsuzaki T, Tsuchiya N, et al. (2008) Associations of interleukin-6 with interleukin-1beta, interleukin-8 and macrophage inflammatory protein-1beta in midlife women. Cytokine 41: 302-306.

287. Chu CJ, Lu RH, Wang SS, Chang FY, Lin SY, et al. (2007) Plasma levels of interleukin-6 and interleukin-8 in Chinese patients with non-alcoholic fatty liver disease. Hepatogastroenterology 54: 2045-2048.

288. Di Sebastiano P, di Mola FF, Di Febbo C, Baccante G, Porreca E, et al. (2000) Expression of interleukin 8 (IL-8) and substance P in human chronic pancreatitis. Gut 47: 423-428.

289. Saurer L, Reber P, Schaffner T, Buchler MW, Buri C, et al. (2000) Differential expression of chemokines in normal pancreas and in chronic pancreatitis. Gastroenterology 118: 356-367.

290. Farrow B, Sugiyama Y, Chen A, Uffort E, Nealon W, et al. (2004) Inflammatory mechanisms contributing to pancreatic cancer development. Ann Surg 239: 763-769; discussion 769-771.

291. Zeh HJ, Winikoff S, Landsittel DP, Gorelik E, Marrangoni AM, et al. (2005) Multianalyte profiling of serum cytokines for detection of pancreatic cancer. Cancer Biomark 1: 259-269.

292. Bloomston M, Zhou JX, Rosemurgy AS, Frankel W, Muro-Cacho CA, et al. (2006) Fibrinogen gamma overexpression in pancreatic cancer identified by large-scale proteomic analysis of serum samples. Cancer Res 66: 2592-2599.

293. Gahne B, Juneja RK, Stratil A (1987) Genetic polymorphism of human plasma alpha 1B-glycoprotein: phenotyping by immunoblotting or by a simple method of 2-D electrophoresis. Hum Genet 76: 111-115.

294. Adkins JN, Varnum SM, Auberry KJ, Moore RJ, Angell NH, et al. (2002) Toward a human blood serum proteome: analysis by multidimensional separation coupled with mass spectrometry. Mol Cell Proteomics 1: 947-955.

295. Letendre SL, Lanier ER, McCutchan JA (1999) Cerebrospinal fluid beta chemokine concentrations in neurocognitively impaired individuals infected with human immunodeficiency virus type 1. J Infect Dis 180: 310-319.

296. Singh L, Bakshi DK, Majumdar S, Vasishta RK, Arora SK, et al. (2007) Expression of interferon-gamma- inducible protein-10 and its receptor CXCR3 in chronic pancreatitis. Pancreatology 7: 479-490.

297. Goecke H, Forssmann U, Uguccioni M, Friess H, Conejo-Garcia JR, et al. (2000) Macrophages infiltrating the tissue in chronic pancreatitis express the chemokine receptor CCR5. Surgery 128: 806-814.

298. Folz RJ, Peno-Green L, Crapo JD (1994) Identification of a homozygous missense mutation (Arg to Gly) in the critical binding region of the human EC-SOD gene (SOD3) and its association with dramatically increased serum enzyme levels. Hum Mol Genet 3: 2251-2254.

299. Saitoh D, Ookawara T, Fukuzuka K, Kawakami M, Sakamoto T, et al. (2001) Characteristics of plasma extracellular SOD in burned patients. Burns 27: 577-581.

300. Adachi T, Yamazaki N, Tasaki H, Toyokawa T, Yamashita K, et al. (1998) Changes in the heparin affinity of extracellular-superoxide dismutase in patients with coronary artery atherosclerosis. Biol Pharm Bull 21: 1090-1093.

301. Shang YX, Cai XX, Han XH, Zhao SQ, Kong SQ, et al. (2003) [Change of neurokinin A plasma level in asthmatic children]. Zhonghua Er Ke Za Zhi 41: 457-459.

302. Bruno G, Tega F, Bruno A, Graf U, Corelli F, et al. (2003) The role of substance P in cerebral ischemia. Int J Immunopathol Pharmacol 16: 67-72.

303. Cho YS, Park SY, Lee CK, Yoo B, Moon HB (2003) Elevated substance P levels in nasal lavage fluids from patients with chronic nonproductive cough and increased cough sensitivity to inhaled capsaicin. J Allergy Clin Immunol 112: 695-701.

304. Akimoto Y, Hirabayashi J, Kasai K, Hirano H (1995) Expression of the endogenous 14-kDa beta-galactoside-binding lectin galectin in normal human skin. Cell Tissue Res 280: 1-10.

305. Niedergethmann M, Wostbrock B, Sturm JW, Willeke F, Post S, et al. (2004) Prognostic impact of cysteine proteases cathepsin B and cathepsin L in pancreatic adenocarcinoma. Pancreas 29: 204-211.

306. Ungefroren H, Voss M, Jansen M, Roeder C, Henne-Bruns D, et al. (1998) Human pancreatic adenocarcinomas express Fas and Fas ligand yet are resistant to Fas-mediated apoptosis. Cancer Res 58: 1741-1749.

307. Suda T, Takahashi T, Golstein P, Nagata S (1993) Molecular cloning and expression of the Fas ligand, a novel member of the tumor necrosis factor family. Cell 75: 1169-1178.

308. Albanese J, Dainiak N (2000) Ionizing radiation alters Fas antigen ligand at the cell surface and on exfoliated plasma membrane-derived vesicles: implications for apoptosis and intercellular signaling. Radiat Res 153: 49-61.

309. Satoh K, Shimosegawa T, Masamune A, Hirota M, Koizumi M, et al. (1999) Fas ligand is frequently expressed in human pancreatic duct cell carcinoma. Pancreas 19: 339-345.

310. Boltze C, Schneider-Stock R, Aust G, Mawrin C, Dralle H, et al. (2002) CD97, CD95 and Fas-L clearly discriminate between chronic pancreatitis and pancreatic ductal adenocarcinoma in perioperative evaluation of cryocut sections. Pathol Int 52: 83-88.

311. Hebert C, Norris K, Della Coletta R, Reynolds M, Ordonez J, et al. (1999) Cell surface colligin/Hsp47 associates with tetraspanin protein CD9 in epidermoid carcinoma cell lines. J Cell Biochem 73: 248-258.

312. Yokota S, Kubota H, Matsuoka Y, Naitoh M, Hirata D, et al. (2003) Prevalence of HSP47 antigen and autoantibodies to HSP47 in the sera of patients with mixed connective tissue disease. Biochem Biophys Res Commun 303: 413-418.

313. Shimokawa Ki K, Katayama M, Matsuda Y, Takahashi H, Hara I, et al. (2002) Matrix metalloproteinase (MMP)-2 and MMP-9 activities in human seminal plasma. Mol Hum Reprod 8: 32-36.

314. Iacobuzio-Donahue CA, Ryu B, Hruban RH, Kern SE (2002) Exploring the host desmoplastic response to pancreatic carcinoma: gene expression of stromal and neoplastic cells at the site of primary invasion. Am J Pathol 160: 91-99.

315. Ellenrieder V, Alber B, Lacher U, Hendler SF, Menke A, et al. (2000) Role of MT-MMPs and MMP-2 in pancreatic cancer progression. Int J Cancer 85: 14-20.

316. Degen M, Brellier F, Schenk S, Driscoll R, Zaman K, et al. (2008) Tenascin-W, a new marker of cancer stroma, is elevated in sera of colon and breast cancer patients. Int J Cancer.

317. Schenk S, Muser J, Vollmer G, Chiquet-Ehrismann R (1995) Tenascin-C in serum: a questionable tumor marker. Int J Cancer 61: 443-449.

318. Pauli C, Stieber P, Schmitt UM, Andratschke M, Hoffmann K, et al. (2002) The significance of Tenascin-C serum level as tumor marker in squamous cell carcinoma of the head and neck. Anticancer Res 22: 3093-3097.

319. Takeda A, Otani Y, Iseki H, Takeuchi H, Aikawa K, et al. (2007) Clinical significance of large tenascin-C spliced variant as a potential biomarker for colorectal cancer. World J Surg 31: 388-394.

320. Suzuki H, Kinoshita N, Imanaka-Yoshida K, Yoshida T, Taki W (2008) Cerebrospinal Fluid Tenascin-C Increases Preceding the Development of Chronic Shunt-Dependent Hydrocephalus After Subarachnoid Hemorrhage. Stroke.

321. Juuti A, Nordling S, Louhimo J, Lundin J, Haglund C (2004) Tenascin C expression is upregulated in pancreatic cancer and correlates with differentiation. J Clin Pathol 57: 1151-1155.

322. Koninger J, Giese T, di Mola FF, Wente MN, Esposito I, et al. (2004) Pancreatic tumor cells influence the composition of the extracellular matrix. Biochem Biophys Res Commun 322: 943-949.

323. David G, Lories V, Decock B, Marynen P, Cassiman JJ, et al. (1990) Molecular cloning of a phosphatidylinositol-anchored membrane heparan sulfate proteoglycan from human lung fibroblasts. J Cell Biol 111: 3165-3176.

324. Schofield KP, Gallagher JT, David G (1999) Expression of proteoglycan core proteins in human bone marrow stroma. Biochem J 343 Pt 3: 663-668.

325. Lories V, Cassiman JJ, Van den Berghe H, David G (1992) Differential expression of cell surface heparan sulfate proteoglycans in human mammary epithelial cells and lung fibroblasts. J Biol Chem 267: 1116-1122.

326. Kleeff J, Ishiwata T, Kumbasar A, Friess H, Buchler MW, et al. (1998) The cell-surface heparan sulfate proteoglycan glypican-1 regulates growth factor action in pancreatic carcinoma cells and is overexpressed in human pancreatic cancer. J Clin Invest 102: 1662-1673.

327. Kornmann M, Ishiwata T, Beger HG, Korc M (1997) Fibroblast growth factor-5 stimulates mitogenic signaling and is overexpressed in human pancreatic cancer: evidence for autocrine and paracrine actions. Oncogene 15: 1417-1424.

328. Takahashi M, Nagaretani H, Funahashi T, Nishizawa H, Maeda N, et al. (2001) The expression of SPARC in adipose tissue and its increased plasma concentration in patients with coronary artery disease. Obes Res 9: 388-393.

329. Guweidhi A, Kleeff J, Adwan H, Giese NA, Wente MN, et al. (2005) Osteonectin influences growth and invasion of pancreatic cancer cells. Ann Surg 242: 224-234.

330. Lang TH, Willinger U, Holzer G (2004) Soluble cathepsin-L: a marker of bone resorption and bone density? J Lab Clin Med 144: 163-166.

331. Dong M, Wang H, Huang H (2007) Alterations of serum cathepsins B and L in pre-eclampsia. Clin Chim Acta 377: 285-287.

332. O'Bryan JP, Fridell YW, Koski R, Varnum B, Liu ET (1995) The transforming receptor tyrosine kinase, Axl, is post-translationally regulated by proteolytic cleavage. J Biol Chem 270: 551-557.

333. Yanagita M, Arai H, Ishii K, Nakano T, Ohashi K, et al. (2001) Gas6 regulates mesangial cell proliferation through Axl in experimental glomerulonephritis. Am J Pathol 158: 1423-1432.

334. Mawby WJ, Holmes CH, Anstee DJ, Spring FA, Tanner MJ (1994) Isolation and characterization of CD47 glycoprotein: a multispanning membrane protein which is the same as integrin-associated protein (IAP) and the ovarian tumour marker OA3. Biochem J 304 (Pt 2): 525-530.

335. Claesson L, Peterson PA (1983) Association of human gamma chain with class II transplantation antigens during intracellular transport. Biochemistry 22: 3206-3213.

336. Roche PA, Teletski CL, Stang E, Bakke O, Long EO (1993) Cell surface HLA-DR-invariant chain complexes are targeted to endosomes by rapid internalization. Proc Natl Acad Sci U S A 90: 8581-8585.

337. Claesson L, Larhammar D, Rask L, Peterson PA (1983) cDNA clone for the human invariant gamma chain of class II histocompatibility antigens and its implications for the protein structure. Proc Natl Acad Sci U S A 80: 7395-7399.

338. Koide N, Yamada T, Shibata R, Mori T, Fukuma M, et al. (2006) Establishment of perineural invasion models and analysis of gene expression revealed an invariant chain (CD74) as a possible molecule involved in perineural invasion in pancreatic cancer. Clin Cancer Res 12: 2419-2426.

339. Gherzi R, Fehmann HC, Eissele R, Goke B (1994) Expression, intracellular localization, and gene transcription regulation of the secretory protein 7B2 in endocrine pancreatic cell lines and human insulinomas. Exp Cell Res 213: 20-27.

340. Cui L, Yu WP, DeAizpurua HJ, Schmidli RS, Pallen CJ (1996) Cloning and characterization of islet cell antigen-related protein-tyrosine phosphatase (PTP), a novel receptor-like PTP and autoantigen in insulin-dependent diabetes. J Biol Chem 271: 24817-24823.

341. Lan MS, Lu J, Goto Y, Notkins AL (1994) Molecular cloning and identification of a receptor-type protein tyrosine phosphatase, IA-2, from human insulinoma. DNA Cell Biol 13: 505-514.

342. Ramjaun AR, Philie J, de Heuvel E, McPherson PS (1999) The N terminus of amphiphysin II mediates dimerization and plasma membrane targeting. J Biol Chem 274: 19785-19791.

343. Capurso G, Lattimore S, Crnogorac-Jurcevic T, Panzuto F, Milione M, et al. (2006) Gene expression profiles of progressive pancreatic endocrine tumours and their liver metastases reveal potential novel markers and therapeutic targets. Endocr Relat Cancer 13: 541-558.

344. Karna E, Surazynski A, Orlowski K, Laszkiewicz J, Puchalski Z, et al. (2002) Serum and tissue level of insulin-like growth factor-I (IGF-I) and IGF-I binding proteins as an index of pancreatitis and pancreatic cancer. Int J Exp Pathol 83: 239-245.

345. Rajaram S, Baylink DJ, Mohan S (1997) Insulin-like growth factor-binding proteins in serum and other biological fluids: regulation and functions. Endocr Rev 18: 801-831.

346. Stone S, Langford K, Seed PT, Khamashta MA, Hunt BJ, et al. (2003) Longitudinal analysis of serum insulin-like growth factor-I and insulin-like growth factor binding protein-1 in antiphospholipid syndrome and in healthy pregnancy. Am J Obstet Gynecol 189: 274-279.

347. Akturk M, Arslan M, Altinova A, Ozdemir A, Ersoy R, et al. (2007) Association of serum levels of IGF-I and IGFBP-1 with renal function in patients with type 2 diabetes mellitus. Growth Horm IGF Res 17: 186-193.

348. Suikkari AM (1989) Insulin-like growth factor (IGF-I) and its low molecular weight binding protein in human milk. Eur J Obstet Gynecol Reprod Biol 30: 19-25.

349. Bell SC, James RF, Jackson JA, Patel SR, Waites GT, et al. (1989) Monoclonal antibodies to human secretory "pregnancy-associated endometrial alpha 1-globulin," an insulin-like growth factor binding protein: characterization and use in radioimmunoassay, Western blots, and immunohistochemistry. Am J Reprod Immunol 20: 87-96.

350. Lee DY, Park SK, Yorgin PD, Cohen P, Oh Y, et al. (1994) Alteration in insulin-like growth factor-binding proteins (IGFBPs) and IGFBP-3 protease activity in serum and urine from acute and chronic renal failure. J Clin Endocrinol Metab 79: 1376-1382.

351. Hansel DE, Rahman A, House M, Ashfaq R, Berg K, et al. (2004) Met proto-oncogene and insulin-like growth factor binding protein 3 overexpression correlates with metastatic ability in well-differentiated pancreatic endocrine neoplasms. Clin Cancer Res 10: 6152-6158.

352. Kim JH, Ho SB, Montgomery CK, Kim YS (1990) Cell lineage markers in human pancreatic cancer. Cancer 66: 2134-2143.
